# Role of Ca^2+^/Calmodulin-Dependent Protein Kinase Type II in Mediating Function and Dysfunction at Glutamatergic Synapses

**DOI:** 10.3389/fnmol.2022.855752

**Published:** 2022-06-20

**Authors:** Archana G. Mohanan, Sowmya Gunasekaran, Reena Sarah Jacob, R. V. Omkumar

**Affiliations:** ^1^Neurobiology Division, Rajiv Gandhi Centre for Biotechnology, Thiruvananthapuram, India; ^2^Research Scholar, Manipal Academy of Higher Education, Manipal, India

**Keywords:** Ca2+/calmodulin-dependent protein kinase type II (CaMKII), glutamatergic synapse, LTP, LTD, synaptic plasticity, CaMKII genetic models, CaMKII mutations

## Abstract

Glutamatergic synapses harbor abundant amounts of the multifunctional Ca^2+^/calmodulin-dependent protein kinase type II (CaMKII). Both in the postsynaptic density as well as in the cytosolic compartment of postsynaptic terminals, CaMKII plays major roles. In addition to its Ca^2+^-stimulated kinase activity, it can also bind to a variety of membrane proteins at the synapse and thus exert spatially restricted activity. The abundance of CaMKII in glutamatergic synapse is akin to scaffolding proteins although its prominent function still appears to be that of a kinase. The multimeric structure of CaMKII also confers several functional capabilities on the enzyme. The versatility of the enzyme has prompted hypotheses proposing several roles for the enzyme such as Ca^2+^ signal transduction, memory molecule function and scaffolding. The article will review the multiple roles played by CaMKII in glutamatergic synapses and how they are affected in disease conditions.

## Introduction

Glutamatergic synapses are the main excitatory synapses in the brain particularly in the cerebral cortex and hippocampus. More than 80% of synapses in the cortex are glutamatergic (Micheva et al., 2010). Glutamatergic transmission plays a major role in neuronal functions in the brain. Imbalances in glutamatergic signaling can lead to several neurodegenerative and psychiatric conditions (Moretto et al., 2018).

Calcium (Ca^2+^) signaling is an essential component in signal transduction at glutamatergic synapses. Calcium signals are tightly regulated since sustained elevation in Ca^2+^ levels can lead to toxicity. In glutamatergic synapses, the spike patterns of Ca^2+^ signals are thought to encode information. Decoding these signals requires the participation of efficient protein machineries that convert them into long-lasting biochemical and cellular changes representing memories. Calcium (Ca^2+^)/calmodulin (CaM)-dependent protein kinase II (CaMKII) at synapses plays a significant role in decoding Ca^2+^ spike patterns and in converting them to corresponding biochemical states. Thus, CaMKII has gained the status of a “memory molecule” by being the initiator of biochemical memory in the brain. However, the multiple isoforms and splice variants of CaMKII that assemble in varying combinations to give rise to several holoenzyme subtypes, makes it so versatile that it is involved in several other functions both in the brain and in other tissues. The phylogenetic relations of CaMKII with other kinases, its structure, its different isoforms and splice variants, biochemical and physiological functions, especially in long-term potentiation (LTP) and long-term depression (LTD), and its role in various diseases have been reviewed recently (Bayer and Schulman, 2019; Giese, 2021; Sloutsky and Stratton, 2021). Its functions specifically in the glutamatergic postsynaptic compartment have also been previously described (Hell, 2014). This article covers the basics on CaMKII including the recent advances in structure, isoforms, activation mechanisms, role in LTP and LTD, regulation of its translation, role in synapse morphology regulation, role in presynaptic mechanisms and role in various pathological conditions with emphasis on its functioning at glutamatergic synapses. *In vivo* models of CaMKII mutants with the associated behavioral phenotypes and CaMKII mutations reported in neurodevelopmental disorders and learning disabilities in humans have also been included in the present review.

## Ca^2+^/Calmodulin-Dependent Protein Kinase Type II Isoforms and Their Function in Glutamatergic Synapses

Even though CaMKII has four distinct isoforms (α, β, γ, and δ) encoded by four different genes with molecular weight ranging from 52 to 83 kDa, α and β are the predominant ones in neurons. CaMKIIα has distinct roles in neuronal plasticity and memory. It is predominant in the hippocampal and in the neocortical areas of the brain. CaMKIIβ is enriched in cerebellum and is involved in neuronal development. While both CaMKIIα and CaMKIIβ are expressed in excitatory pyramidal neurons in the cortex and hippocampus, only CaMKIIβ is found in inhibitory interneurons in these regions (Nicole and Pacary, 2020). CaMKIIδ isoform participates in long-lasting memory storage in the hippocampus (Zalcman et al., 2018, 2019). CaMKIIγ isoform is attributed with the main function of synapse-to-nucleus communication, conveying Ca^2+^ signals to the nucleus and regulating gene expression that is essential for neural plasticity involved in memory (Ma et al., 2014; Cohen et al., 2018).

## Ca^2+^/Calmodulin-Dependent Protein Kinase Type II Structure in Relation to Its Function

**FIGURE 1:**
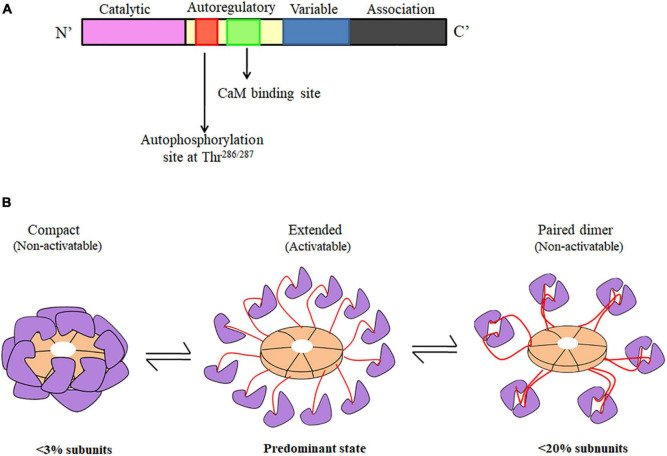
**(A)** Linear representation of CaMKII structure showing catalytic, autoregulatory, variable and association domains. **(B)** CaMKII holoenzyme structure in three different forms-CaMKII can exist predominantly in the activatable state with an extended conformation along with some non-activatable states, which are represented as both compact form and kinase domain paired form. The different subunits of a single CaMKII holoenzyme can exist in any combination of the three forms. Purple color indicates kinase domain, peach color denotes association domain, and red color indicates regulatory domain (Myers et al., 2017).

CaMKII forms large homo or hetero oligomeric assemblies of either single or multiple isoforms (Hoelz et al., 2003; Bayer and Schulman, 2019). The core sequence for the CaMKII isoforms includes an N-terminal catalytic domain, followed by a CaM binding autoregulatory domain containing Thr^286^/Thr^287^, a variable domain that is subject to alternative splicing and a C-terminal self-association domain. A linear representation of a CaMKII subunit is shown in Figure 1A.

**FIGURE 2:**
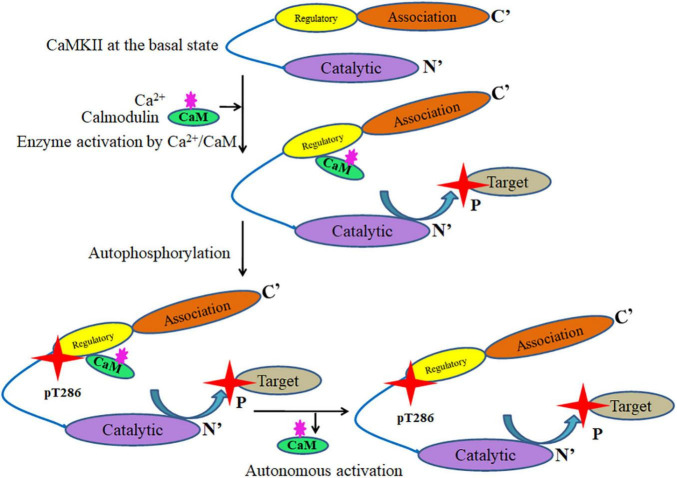
Basic activation mechanism of CaMKII that leads to autonomy resulting from Thr^286^ autophosphorylation. Under basal conditions, the enzyme is present in an autoinhibited state with the regulatory segment bound to the catalytic domain. This can be activated by the binding of Ca^2+^/CaM to the regulatory domain which releases the regulatory segment from the catalytic domain. The activated enzyme can autophosphorylate at Thr^286^ or any other substrates. The autonomous CaMKII thus generated can be catalytically active even in the absence of Ca^2+^/CaM.

Under basal state, the enzyme is present in an autoinhibited state with the regulatory segment bound to the substrate-docking groove on each kinase domain. It can be activated by the binding of Ca^2+^/CaM to the autoregulatory domain which releases the regulatory segment off the kinase domain. Activation of adjacent subunits can result in trans-autophosphorylation at Thr^286^ site (Rich and Schulman, 1998) in the regulatory segment which generates ‘autonomous’ kinase activity even after the initial Ca^2+^-stimulus subsides (Miller and Kennedy, 1986) by preventing the regulatory segment binding on the kinase domain. This inter-subunit autophosphorylation mechanism enables Ca^2+^-spike frequency-detection by CaMKII (De Koninck and Schulman, 1998). The autophosphorylation at Thr^286^ can also increase the affinity of the enzyme for CaM by about 1000-fold, a process termed as CaM trapping. A single autophosphorylated subunit can also rapidly phosphorylate its neighbors. Thus, a brief Ca^2+^ stimulus in the synapse can lead to the persistence of Thr^286^-autophosphorylated CaMKII that represents molecular memory (Figure 2). Autophosphorylation at Thr^286^ is an essential event in the induction of LTP that underlies memory formation.

Once Ca^2+^/CaM dissociates from the kinase, *cis*-autophosphorylation occurs at the CaM binding domain of CaMKII at the Thr^305/306^ position. Phosphorylation at these sites, termed as “inhibitory” or “burst” autophosphorylation, prevents the binding of Ca^2+^/CaM and hence kinase cannot be further stimulated. Autophosphorylation at Thr^305^ and Thr^306^ before phosphorylation of Thr^286^ makes the kinase non-responsive to Ca^2+^/CaM stimulus and such a kinase cannot be activated. On the other hand, if Thr^286^ gets autophosphorylated first, it leads to a holoenzyme in which Thr^305^ and Thr^306^ are protected by Ca^2+^/CaM and cannot be phosphorylated (Bhattacharyya et al., 2020). It is also reported that CaMKII phosphorylation at Thr^305/306^ is selectively promoted by LTD inducing stimuli and not by LTP inducing stimuli, and phosphorylation at Thr^305/306^ directs CaMKII movement during LTD from excitatory to inhibitory synapses. This phosphorylation can also reduce the activity of phospho-Thr^286^ CaMKII in the absence of Ca^2+^ (Cook et al., 2021).

The first snapshot of the 3D structure of this enzyme was an electron microscopy (EM) image of CaMKII purified from rabbit skeletal muscle (Woodgett et al., 1983) that revealed a symmetrical hexagonal structure, composed of two stacked 6-membered rings. Since then, several hypotheses have been proposed about its structure in relation to its function. The catalytic/autoregulatory domains of each subunit are attached to the hexameric ring by a stalk-like appendage that presumably allows subunits to behave independently of one another for activity and Ca^2+^/CaM binding, but in concert with one another for the intra-holoenzyme autophosphorylation reaction (Figure 1B). Most of the crystallographic studies provided structures at atomic resolution of truncated forms having single or multiple domains (Hoelz et al., 2003; Rosenberg et al., 2006) giving insights on the mechanism of catalytic activity and atomic level details of the interactions holding the 3D structure and interactions between domains.

The recent studies based on single-particle EM (Myers et al., 2017; Bhattacharyya et al., 2020) in combination with biochemical and live-cell imaging experiments (Buonarati et al., 2021) further substantiated the multimeric structure of CaMKII holoenzyme having a rigid central hub complex formed by the association domains. The kinase domains are linked to the hub by the intrinsically disordered and highly flexible linker regions (residues 301–344) so that they can readily perform inter-subunit autophosphorylation. The holoenzymes range from 15–35 nm in diameter. This model also predicts that CaMKII holoenzymes can exist in three different conformations. Among these three conformations, <3% of the holoenzymes are in the compact conformation, ∼20% appears to form kinase dimers and most of the kinase domains are ordered independently both *in vitro* and inside the cells. CaMKII holoenzymes which appear as either compact or kinase dimers are inactive, whereas the fraction with fully extended kinase domains is in the activatable state (Figure 1B; Myers et al., 2017; Bayer and Schulman, 2019).

The formation of extended intra-holoenzyme kinase dimers could enable cooperative activation by CaM in both α and β isoforms (Myers et al., 2017; Bhattacharyya et al., 2020; Buonarati et al., 2021) but there could be distinct steric positioning of kinase domains in the CaMKIIα versus β holoenzyme due to differences in the linker length. This explains the differences in the autophosphorylation states of both the isoforms; CaMKIIα acquires Thr^286^ phosphorylation more readily than Thr^305/306^ phosphorylation whereas inhibitory autophosphorylation at Thr^306/307^ in CaMKIIβ occurs more readily. Inter-holoenzyme kinase dimer formation is thought to involve a high order clustering among CaMKII holoenzymes and is present in minimal quantities under normal physiological conditions for both the isoforms. But it is enhanced in both excitotoxic and ischemic conditions and the high-order CaMKII clustering formed by inter-holoenzyme kinase domain dimerization is reduced for the β isoform for both basal and excitotoxicity-induced clusters, both *in vitro* and in neurons (Buonarati et al., 2021). Much of the studies on holoenzyme structure have been carried out using homomers of either α or β isoforms. However, heteromultimeric CaMKII formed by α and β is known to play key functions in the brain. Structural insights into heteromultimeric forms of CaMKII would help in further advancing the understanding of the physiological functioning of this enzyme. It has been also noted that a small percentage (<4%) of holoenzymes of CaMKIIα were found as 14-mers even with full-length kinase domains (Myers et al., 2017) whereas CaMKIIβ can even exist in 16-mers (Buonarati et al., 2021). The existence of a full-length 14-mer is thought to be an intermediate state in which the exchange of subunits is possible (Myers et al., 2017) and it entails the exchange of activated subunits between two activated, or an activated and a non-activated holoenzyme (Bhattacharyya et al., 2020). This hypothesis was supported by the finding that proteolytic cleavage of the kinase domains from a 12-meric holoenzyme preparation results in the subsequent formation of 14-meric hub domain assemblies (Rosenberg et al., 2006). The function of this kind of subunit exchange is currently unknown, but it is speculated that it can be a part of repair mechanisms of individual subunits and/or synaptic plasticity mechanisms (Bayer and Schulman, 2019).

## Ca^2+^/Calmodulin-Dependent Protein Kinase Type II Activation in Response to Ca^2+^ Influx Through *N*-Methyl-*D*-Aspartate Receptor

*N*-Methyl-D-aspartate receptor (NMDAR) is an ionotropic glutamate receptor with high Ca^2+^ permeability that plays an important role in excitatory neurotransmission in the central nervous system (CNS). Glutamate binding to α-amino-3-hydroxy-5-methyl-4-isoxazole propionic acid receptors (AMPARs) can induce depolarization in the postsynaptic membrane of glutamatergic synapses. The binding of glutamate and glycine and the depolarization-induced removal of Mg^2+^ block causes NMDAR to open and conduct Ca^2+^ and Na^+^ into the cell. This Ca^2+^ influx activates several important signaling pathways involving different protein kinases including CaMKII and phosphatases. Activated CaMKII can bind to various membrane proteins as listed in Table 1. The enzyme can interact with each of these proteins either in the Ca^2+^/CaM activated form or in the autophosphorylated form. It can bind with high affinity at the GluN2B subunit of NMDAR and phosphorylate GluN2B-Ser^1303^ (Omkumar et al., 1996). GluN2B-binding can also happen at the T-site of CaMKII (site where Thr^286^ is bound in the inactive state) making the enzyme permanently active even after the Ca^2+^ stimulus subsides (Bayer et al., 2001). In addition, the kinetic parameters of CaMKII activity and its affinity for ATP are altered in an allosteric manner upon binding to GluN2B (Pradeep et al., 2009; Cheriyan et al., 2011; Madhavan et al., 2020) and this regulation is limited only to the subunit of the enzyme that binds GluN2B (Cheriyan et al., 2012). CaMKII activated in the cytosol can translocate to the postsynaptic membrane where the NMDAR complex is embedded in the postsynaptic density (PSD). CaMKII reversibly translocates to synaptic sites in response to brief stimuli, but its resident time at the synapse increases after longer stimulation (Bayer et al., 2006). It is also reported that the phosphorylation status of GluN2B at Ser^1303^ also regulates GluN2B-CaMKII interaction (Raveendran et al., 2009), whereas the phosphorylation status of Ser^1303^, in turn, is regulated by the action of kinases and phosphatases (Ramya et al., 2012). In the GluN2B-bound state, the enzyme becomes resistant to the action of phosphatases (Cheriyan et al., 2011) indicating GluN2B-induced structural changes which can be abolished by specific mutations in CaMKII (Mayadevi et al., 2016). This could be a possible reason for the resistance of phospho-Thr^286^-CaMKIIα to phosphatases in the PSD (Mullasseril et al., 2007). Autonomy of CaMKII due to GluN2B-binding can be terminated only by dissociation of CaMKII from GluN2B. Repeated Ca^2+^ influx through NMDAR promotes the persistent binding of CaMKII to GluN2B which occurs during LTP (Bayer et al., 2006).

**TABLE 1:** Protein ligands of CaMKII in the postsynaptic compartment of glutamatergic synapses.

Sl. No.	Protein ligand to which CaMKII binds	Region of binding	Functional implications of this binding	Reference(s)
1	NMDAR subunit GluN2B	839–1120	The binding requires auto phosphorylated CaMKII; tethering at the synaptic membrane; LTP	Lisman et al., 2012
2	NMDAR subunit GluN2B	1289–1310	Activated CaMKII can bind; tethering at the synaptic membrane; LTP	Lisman et al., 2012
3	NMDAR subunit GluN2A	1349–1464	Synaptic targeting	Gardoni et al., 1999
4	NMDAR subunit GluN1	845–863	Synaptic targeting	Leonard et al., 2002
5	Cav1.2	1589–1690	Tethering at the synaptic membrane	Hudmon et al., 2005
6	Densin-180	1247–1495	Membrane localization	Strack et al., 2000b; Robison et al., 2005
7	Tiam 1	1540–1560	Constitutive CaMKII activation; LTP	Saneyoshi et al., 2019
8	Ether-a-go-go (Eag)	731–803	Constitutive CaMKII activation; LTP	Sun et al., 2004

### Long Term Potentiation Induction by the Activation of *N*-Methyl-D-Aspartate Receptors-Role of Ca^2+^/Calmodulin-Dependent Protein Kinase Type II in *N*-Methyl-D-Aspartate Receptor-Dependent Long Term Potentiation

LTP is a process in which brief periods of synaptic activity produces long-lasting increase in the strength of a synapse, as shown by an increase in the size of the excitatory postsynaptic current (EPSC) (Lisman et al., 2012; Bliss and Collingridge, 2019). Several studies have shown that LTP has the essential characteristics of a cellular mechanism that could underlie memory and can serve as an excellent cellular model of memory. Impairment in LTP formation predicts memory impairment in human subjects (Di Lorenzo et al., 2020). LTP occurring at CA3-CA1 synapses (between Schaffer collateral (SC) terminals and CA1 pyramidal neurons) of the hippocampal region is mainly mediated through NMDARs and occurs predominantly by postsynaptic modifications (MacDonald et al., 2006). This model of LTP is a suitable model for associative learning (Baltaci et al., 2019).

LTP has an early phase which is independent of protein synthesis, called early-LTP (E-LTP), and a late phase (L-LTP) which involves the activation of transcription factors and is dependent on protein synthesis, during which structural changes are observed. Single brief tetanus leads to E-LTP that lasts up to 1–3 h and intermittent and repetitive stimulations (or single stronger tetanus) produce L-LTP that lasts at least 24 h (Baltaci et al., 2019). During the induction of LTP, Ca^2+^ influx through NMDARs activates signaling pathways that lead to synaptic modifications (Malenka et al., 1989). NMDAR-dependent LTP requires one or more trains of 100 Hz stimulations (Baltaci et al., 2019).

**FIGURE 3:**
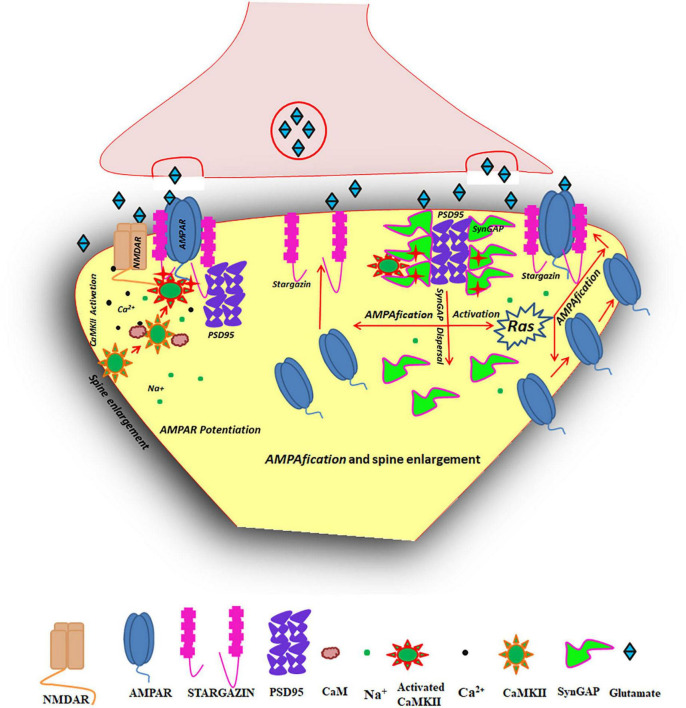
Schematic diagram shows the role of CaMKII in LTP. CaMKII activity at the PSD is essential for the induction and maintenance of LTP, either through (i) enhancement of AMPAR conductance or through (ii) AMPAfication of the postsynaptic site. In either of these functions, activation of CaMKII along with its translocation to its own adapters at the PSD, especially to the GluN2B subunit of NMDAR is essential. The translocated CaMKII can phosphorylate its substrates involved in the induction and maintenance of LTP. (i) AMPAR potentiation-The phosphorylation at Ser^831^ of GluA1 of AMPAR by CaMKII enhances the single channel conductance of AMPAR especially AMPAR formed by GluA1 homomers (Derkach et al., 1999). (ii) AMPAfication (conversion of silent synapses to active synapses)- AMPARs are positioned in the PSD by interaction with many proteins, especially stargazin. Phosphorylation of stargazin by CaMKII results in its dissociation from lipid rafts and binding to PSD95 to make more AMPAR slots on the membrane (slot hypothesis for AMPAfication). In addition to this, CaMKII can phosphorylate SynGAP which results in its elimination from the synapse followed by the activation of Ras/ERK signaling which mediates AMPAfication or AMPAR recruitment to the PSD. These signaling cascades finally lead to spine enlargement.

Over three decades of study suggests that CaMKII is one of the key players in LTP (Zalcman et al., 2018). Inhibition of CaMKII activity blocks the induction as well as maintenance of LTP (Malenka et al., 1989; Malinow et al., 1989; Tao et al., 2021). In response to sufficient influx of Ca^2+^ into the postsynaptic neuron, CaMKII gets activated by the binding of Ca^2+^/CaM and autophosphorylated at Thr^286^. Both these forms of CaMKII can translocate to PSD and bind to GluN2B. Autonomously active nature of Thr^286^ phosphorylated CaMKII as well as GluN2B-bound CaMKII is proposed to contribute toward molecular memory. But Thr^286^ autophosphorylation does not have an essential role in NMDAR dependent synaptic potentiation in early postnatal development and in adult dentate gyrus, where neurogenesis occurs (Giese, 2021). Persistent nature of GluN2B-CaMKII interaction could also contribute towards its role in maintaining synaptic strength (Sanhueza et al., 2011). If this interaction is impaired by mutations on the binding sites on CaMKII and/or GluN2B (Yang and Schulman, 1999; Strack et al., 2000a; Mayadevi et al., 2002; Pradeep et al., 2009), then LTP gets impaired (Barria and Malinow, 2005). The binding of GluN2B locks CaMKII in an active conformation and the enzyme can phosphorylate its substrates present in the PSD. The protein substrates of CaMKII in the PSD and the physiological consequences of their phosphorylation status are listed out in Supplementary Table 1 (McGlade-McCulloh et al., 1993; Inagaki et al., 1997; Gardoni et al., 2003, 2006; Oh et al., 2004; Chen and Roche, 2007; Shin et al., 2012; Zhang et al., 2019; Zybura et al., 2020). One of the main effectors of LTP is AMPAR. CaMKII that is localized in PSD through interaction with GluN2B can phosphorylate Ser^831^ residue of the GluA1 subunit of AMPAR causing potentiation of the single channel conductance of AMPAR (Figure 3; Barria et al., 1997a,b). As part of LTP, more AMPARs are recruited to the synapses and this process is called *AMPAfication* (Malenka and Nicoll, 1999). The process of *AMPAfication* makes the transmission even stronger (Zhu and Malinow, 2002). It is also reported that the interaction of CaMKII with GluN2B effects a liquid-liquid phase separation with co-segregation of AMPA receptors and the synaptic adhesion molecule neuroligin into a phase-in-phase assembly indicating the formation of functional nanodomains in the synapse (Hosokawa et al., 2021).

Other than AMPAR, CaMKII has other downstream targets such as transmembrane AMPAR-regulatory proteins (TARPs). TARPs are auxiliary proteins that help in AMPAR functions and trafficking (Jackson and Nicoll, 2011). They have several phosphorylation sites for CaMKII which are implicated in the positioning and trapping of AMPAR in PSD (Supplementary Table 1). The C-tail of the TARP family member, stargazin (TARPγ-2) can be phosphorylated by CaMKII which disrupts the interaction of stargazin with phospholipids in the membrane and eventually allows stargazin to bind with PSD95, a major scaffolding protein in PSD to which many other proteins can bind. In this way, stargazin can trap AMPAR in the PSD (Opazo et al., 2010; Hafner et al., 2015). It is also known that the hippocampally enriched TARPγ-8, but not TARPγ-2/3/4, is a critical CaMKII substrate necessary for LTP induction. The residues of TARPγ-8, Ser^277^ and Ser^281^ are major phosphorylation sites for CaMKII, which sufficiently enhances AMPAR transmission. Mutations of these residues impair LTP, without affecting AMPAR-mediated basal transmission and protein levels of AMPAR in PSD or extrasynaptic regions (Park et al., 2016).

CaMKII can also trap AMPAR in the postsynaptic site through other pathways such as Ras/ERK signaling. In the postsynaptic site, SynGAP (synaptic Ras/Rap GTPase-activating protein) is highly enriched and harbors phosphorylation sites for CaMKII. SynGAP contains C-terminal PDZ binding domain which interacts with PSD95 under basal conditions. During LTP induction, CaMKII can phosphorylate this protein. This phosphorylation decreases the affinity of SynGAP toward PSD95, which in turn dissociates away from the same. The massive removal of SynGAP makes more PSD95 available for binding of TARPs and thereby AMPAR trapping in the postsynaptic site (Figure 3; Gamache et al., 2020).

The synaptic SynGAP dispersion also decreases its RasGAP activity, leading to the activation of Ras/ERK signaling crucial for AMPAR delivery (Walkup et al., 2016). The phosphorylation of SynGAP by CaMKII leads to activation of Ras/ERK signaling and inactivation of Rap. The activation of Ras/ERK signaling drives AMPAR delivery to the postsynaptic site whereas Rap mediates AMPAR removal upon its activation. Thus, SynGAP phosphorylation by CaMKII can enhance AMPAR recruitment during LTP (Zhu et al., 2002; Rumbaugh et al., 2006; Wang C.C. et al., 2013; Araki et al., 2015; Walkup et al., 2015).

LTP is also accompanied by increase in spine volume mediated by activation of CaMKII. Activated CaMKII can influence the activity of Rho GTPase–regulatory proteins [e.g., RhoGEFs (guanine nucleotide exchange factors that activate Rho GTPases) and/or RhoGAPs (GTPase-activating proteins that inhibit Rho GTPases)] to promote actin polymerization in the head and neck region of dendritic spines (Herring and Nicoll, 2016). This results in an increase in size of the spine head and diameter of the neck. Increased actin polymerization also results in the reorganization of PSD proteins in such a way that more AMPARs can be incorporated. SynGAP dispersion from PSD resulting from CaMKII phosphorylation is also related to spine enlargement (Araki et al., 2015).

LTP induction is also associated with the rapid formation of a positive feedback loop, formed by a reciprocally activating kinase-effector complex (RAKEC) in dendritic spines, which consist of CaMKII and Tiam1, a Rac1-specific guanine nucleotide exchange factor (Rac-GEF). Activated CaMKII can persistently interact with Tiam1, in stimulated spines enabling the persistence and confinement of a molecular memory (Saneyoshi et al., 2019). The constitutive activation of CaMKII by occupation of its T-site would help to maintain Tiam1 phosphorylation even after Ca^2+^ concentration subsides. This mechanism can therefore convert transient Ca^2+^ signaling into a persistent activation of Rac1 (protein required for spine formation and enlargement) and its downstream actin regulators. This pathway helps in the maintenance of the enlarged spine and thereby contributes to structural LTP (Kojima et al., 2019).

NMDAR activation in pyramidal neurons causes CaMKII-dependent phosphorylation of the guanine-nucleotide exchange factor (GEF), kalirin-7 at residue Thr^95^, regulating its GEF activity, leading to activation of Rac1 and rapid enlargement of existing spines. Kalirin-7 also interacts with AMPA receptors and controls their synaptic expression (Xie et al., 2007).

During LTP maintenance, the levels of protein kinase M zeta (PKMζ), a constitutively active protein kinase C (PKC) isoform, are elevated. PKMζ maintains synaptic potentiation by preventing AMPAR endocytosis and promoting stabilization of dendritic spine growth. Inhibition of PKMζ, with zeta-inhibitory peptide (ZIP), can reverse LTP and impair established long-term memories (LTMs). Activated CaMKII can release the translational block on PKMζ mRNA, thereby helping in long-term maintenance of LTP (Patel and Zamani, 2021). It has been shown by direct evidence that CaMKII is essential for memory storage (Rossetti et al., 2017) by using a kinase-dead mutant (K42M) in the hippocampus where the mutant disrupted CaMKII signaling *in vivo*.

#### Putative Mechanisms of Memory Storage by Ca^2+^/Calmodulin-Dependent Protein Kinase Type II

While considerable insights have been obtained on the mechanisms by which LTP-inducing tetanic stimuli are converted to enhanced AMPAR activity at the postsynaptic side, the mechanisms by which the potentiated state is maintained has been intensively debated (Giese et al., 1998; Buard et al., 2010; Coultrap et al., 2012; Chang et al., 2017; Giese, 2021; Tao et al., 2021). Even long-lasting structural changes such as spine enlargement are maintained by dynamic molecular mechanisms (Gamache et al., 2020). Among the several molecular systems that were proposed to sustain altered biochemical states, the bistable switch model involving CaMKII (Lisman and Zhabotinsky, 2001) has attracted considerable attention, in which the unphosphorylated and Thr^286^-phosphorylated states of CaMKII represented the “OFF” and “ON” states respectively. The ability of the CaMKII oligomer to sustain its autophosphorylated state by autonomous activity has initially been proposed to convert information encoded in Ca^2+^-spikes into stable biochemical traces (Miller and Kennedy, 1986; Hudmon and Schulman, 2002). However, rigorous computational modeling studies showed that successful functioning of the switch requires the participation of protein phosphatase 1 (PP1) and GluN2B (Miller et al., 2005; Michalski, 2013; Urakubo et al., 2014; Lisman and Raghavachari, 2015). The switch was predicted to function in an energy-efficient manner and remain active despite protein turnover (Lisman and Zhabotinsky, 2001). In the unpotentiated synapse, the switch will be in the “OFF” state with CaMKII mostly unphosphorylated. Any phosphorylation supported by resting Ca^2+^ concentration will be successfully annihilated by PP1–mediated dephosphorylation thereby preventing a slow drift to the autophosphorylated “ON” state thus providing stability to the “OFF” state.

LTP-inducing stimulus causes extensive CaMKII autophosphorylation at Thr^286^ due to high Ca^2+^ levels. Autophosphorylated CaMKII that translocates to the PSD will be more than sufficient to saturate the available PP1 activity. Thus, autophosphorylated CaMKII would compete out PP1 activity and thus the high level of autophosphorylation and autonomous activity will be maintained thereby giving stability to the “ON” state. Continued phosphorylation required to negate the effect of PP1 activity while maintaining the “ON” state, leads to consumption of energy in the form of ATP. The model predicted the switch to function in an energy-efficient manner with minimal consumption of ATP and remain active despite protein turnover (Lisman and Zhabotinsky, 2001). Evidence obtained later was in accordance with these predictions on the final functional outcome of the switch, although it involved additional mechanisms than the predicted ones. Accordingly, the revised model (Lisman and Raghavachari, 2015) predicts that energy efficiency is achieved by the reduced dephosphorylation rate of the GluN2B-bound CaMKII (Cheriyan et al., 2011; Mayadevi et al., 2016). Stability against protein turnover is possible since protein turnover operates by subunit exchange between holoenzymes. Thus, replacement of a phosphorylated subunit with a new, unphosphorylated subunit will be followed by phosphorylation of the newly recruited subunit by adjacent autonomous subunits (Stratton et al., 2014; Lisman and Raghavachari, 2015).

In its “ON” state, the switch can initiate and maintain long-term strengthening of the synapse by the multiple mechanisms described above (see section entitled “LTP Induction by the Activation of NMDARs-Role of CaMKII in NMDAR-Dependent LTP”). But later studies indicated that the autophosphorylation of CaMKIIα was required only for rapid learning especially induced by a single stimulus but was not essential for memory formed by multiple trial learning (Irvine et al., 2005, 2011). This was further supported by the evidence that autophosphorylation at Thr^286^ lowers the stimulation frequency required to induce synaptic plasticity and permits CaMKII to better integrate Ca^2+^ signals at physiologically relevant frequencies that would happen only in LTP induction and not in maintenance (Chang et al., 2017). These findings are not consistent with the bistable switch model in which Thr^286^ autophosphorylation is an essential element. These studies suggest that Thr^286^ autophosphorylation might have a major role in the initial capture of information encoded in the synaptic Ca^2+^ spikes with more efficiency. However, inhibition of CaMKII activity can erase LTP showing the involvement of CaMKII in LTP maintenance, further suggesting that CaMKII acts as a molecular storage device (Tao et al., 2021).

CaMKII activity necessary for LTP maintenance at resting Ca^2+^ concentrations could be arising from the autonomous forms of CaMKII, Thr^286^-phosphorylated or GluN2B-bound. If Thr^286^ is dispensable (Irvine et al., 2005, 2011; Chang et al., 2017) as mentioned above, the GluN2B-bound form of CaMKII could provide the autonomous activity. However, in the PSD, all the CaMKII subunits in a holoenzyme need not be bound by GluN2B unlike the *in vitro* experiments (Bayer et al., 1999) in which all CaMKII subunits could be bound by GluN2B. Whether the autonomous activity of the GluN2B-bound subunits of CaMKII in the PSD would be sufficient to maintain LTP needs further investigation, since GluN2B-binding does not spread to other subunits of a holoenzyme of CaMKII like Thr^286^ autophosphorylation.

## Regulation of Translation of Ca^2+^/Calmodulin-Dependent Protein Kinase Type II in Synaptic Plasticity

Gene expression needed for long-lasting synaptic plasticity is tightly regulated. In particular, protein synthesis, regulation of mRNA transport and mRNA stability contribute to the control of gene expression. mRNA translation happens in synaptic locations - dendrites and dendritic spines, which are filled with polyribosomes, translation factors, and mRNAs (Steward and Levy, 1982; Crino and Eberwine, 1996; Job and Eberwine, 2001; Steward and Schuman, 2001).

### Regulation of Ca^2+^/Calmodulin-Dependent Protein Kinase Type II by Cytoplasmic Polyadenylation Element-Binding Protein in Long Term Potentiation

Cytoplasmic polyadenylation element (CPE) present in the 3′ untranslated region (UTR) of mRNAs plays a major role in the regulation of translation in response to cellular signals (Klann and Dever, 2004). CPE sequence present in CaMKIIα mRNA helps in its rapid translation during LTP (Ouyang et al., 1997; Giovannini et al., 2001).

The neuronal CPE-binding protein (CPEB) protein from *Aplysia* has an amino-terminal extension, which can be converted into a prion-like molecule and this mechanism will aid in sustained protein synthesis. Thus, this process would play crucial roles during synaptic plasticity (Si et al., 2003). CPEB blocks translation when it is bound to CPE. Upon phosphorylation, CPEB can dissociate from CPE thereby triggering a series of molecular events leading to initiation of translation. CPEB can be phosphorylated by CaMKII (Wu et al., 1998). CPE-mediated translation following membrane depolarization is also CaMKII-dependent (Lisman et al., 2002). The 3′UTR of CaMKII and other specific mRNAs bind CPEB and polyadenylation specificity factor (CPSF) leading to translational arrest. With NMDAR activation, aurora kinase and CaMKII get activated leading to phosphorylation of CPEB. This is followed by CPEB-CPSF interaction which allows poly(A) polymerase (PAP) recruitment to this complex. PAP initiates the poly(A) tail elongation. This in turn activates poly(A)-binding protein (PABP) which binds to poly(A) tail and initiates interaction with elongation factor eIF4G and thereby activates translation.

Hence, CaMKII activation after LTP activates the CPE-dependent translation which in turn translates CaMKIIα mRNA. This feedforward mechanism is very important for maintaining sustained protein synthesis in LTP and memory (Klann and Dever, 2004).

### Regulation of Ca^2+^/Calmodulin-Dependent Protein Kinase Type II by Elongation Factors in Long Term Potentiation

Translation can be regulated even at the elongation level via phosphorylation of the eukaryotic elongation factor 2 (eEF2), which is a GTP binding protein (Moldave, 1985). eEF2 kinase is regulated by mammalian target of rapamycin (mTOR) activation, which phosphorylates the eEF2 kinase near the CaM binding site, resulting in decreased kinase activity (Browne and Proud, 2004).

In dendrites of cultured cortical neurons (Marin et al., 1997) and tadpole tecta (Scheetz et al., 1997), NMDAR activation leads to phosphorylation of the eEF2 factor thus leading to elongation becoming a rate-limiting step in translation. This is correlated with increased CaMKIIα synthesis but decrease in overall protein synthesis (Scheetz et al., 2000). Similarly, chemically-induced LTP also leads to increased eEF2 phosphorylation with decreased protein synthesis, but with increase in Arc and Fos protein levels (Chotiner et al., 2003). So, phosphorylation of eEF2 leads to overall decrease in protein synthesis but with exceptions of increased translation like that of CaMKIIα (Scheetz et al., 2000).

## Regulation of Neuromodulator Release by Ca^2+^/Calmodulin-Dependent Protein Kinase Type II

The neurotrophins (NTs) are involved as major players in synaptic development and synaptic plasticity (Poo, 2001). Among the NTs – Neuregulin (NRG), BDNF, NT-3 and NT-4, extensive research has been done on BDNF and its role in synaptic plasticity. Postsynaptic NMDAR gating is regulated by BDNF signaling (Levine et al., 1995, 1998). BDNF is important in LTP, as seen by lack of proper establishment of LTP in BDNF knockout (KO) mouse models (Korte et al., 1995; Patterson et al., 1996). BDNF supports high-frequency transmitter release, which is required for LTP induction (Figurov et al., 1996; Gottschalk et al., 1998; Pozzo-Miller et al., 1999; Abidin et al., 2006).

Moro et al. (2020) reported reduced BDNF secretion in mouse deficient in α and β CaMKII [αβCaMKII double-knockout (DKO)] hippocampal neurons. These neurons had drastically reduced levels of BDNF and fewer BDNF containing dense core vesicles (DCV) targeted to the axon, leading to fewer DCVs per synapse and thus reduced BDNF secretion upon stimulation. CaMKIIβ is crucial for increasing the amount of secreted BDNF by CaMKIV and phospho-cAMP-response element binding protein (CREB) pathway. Interestingly, active CaMKIIβ and not CaMKIIα or inactive CaMKIIβ/CaMKIIα could restore the reduced levels of BDNF expression (Moro et al., 2020). BDNF binds to TrkB and this activates CaMKIIβ further leading to a series of downstream signaling events. Subsequently, Ca^2+^/CaM enters into the nucleus and CaMKIV gets activated, phosphorylating CREB at Ser^133^ position, along with nuclear-localized neurogranin. Phosphorylated CREB promotes BDNF transcription (Wheeler et al., 2008; Ma et al., 2014; Wang et al., 2017). Thus, BDNF-mediated activation of CaMKIIβ acts as a positive feedback loop to initiate the expression of the neuromodulator (Moro et al., 2020).

## Ca^2+^/Calmodulin-Dependent Protein Kinase Type II in Axonal/Dendritic Growth Regulation Promoting Synaptic Strength

### Role of Ca^2+^/Calmodulin-Dependent Protein Kinase Type II α

Alterations in synaptic strength are brought about majorly through post-translational modifications such as phosphorylation or dephosphorylation of synapse associated proteins (Davis and Squire, 1984; Yan-You Huang et al., 1996). Miller et al. showed that mutating the targeting signal at the 3′UTR of CaMKIIα mRNA caused significant reduction in the level of CaMKIIα in PSDs and impairments in L-LTP and LTM. The 3′UTR mutants in BL6 mice showed poor behavioral performances in fear conditioning, water maze and object recognition indicating cognitive alterations (Miller et al., 2002).

Wu et al. (1998) and Wells et al. (2001) demonstrated that dendritic CaMKIIα is inducible by showing an increase in CaMKIIα in synaptosomes prepared from the visual cortex of dark-reared rat pups that were transferred to light. Tetanic stimulation was found to increase CaMKIIα levels in stratum radiatum of CA1 (Ouyang et al., 1999), which suggests that CaMKIIα present in PSDs, might arise from the activity-dependent translation of dendritic mRNAs. Assembly of CaMKII holoenzymes occur after the translation of the subunits. The β subunit facilitates the association of the holoenzyme with actin cytoskeleton and thereby localization to the synapses (Shen et al., 1998). Since the mRNA of β subunit is located only in the soma (Burgin et al., 1990), some of the CaMKIIα might be transported into dendrites as pre-assembled holoenzyme (Miller et al., 2002).

Miller et al. (2002) also showed that disrupting the dendritic localization of CaMKIIα mRNA disrupted LTM but not short-term memory (STM) formation. Hence, dendritic CaMKIIα might be a requirement for memory consolidation. Local CaMKIIα synthesis might facilitate transmission by regulating AMPAR phosphorylation (Barria et al., 1997b) or by inserting additional AMPARs into the synapse (Hayashi et al., 2000). CaMKIIα has also been reported to be stabilizing the dendritic arbors and thus regulating synapse shape and density (Wu and Cline, 1998; Koh et al., 1999; Rongo and Kaplan, 1999). Filopodia-like extensions and movements in the dendritic arbors play an important role for neurons in order to determine new contact sites, which can then evolve into nascent synapses and mature into functional synaptic connections (Vaughn, 1989; Jontes and Smith, 2000; Wong and Wong, 2000; Ahmari and Smith, 2002). For all these mechanisms, continued supply of CaMKIIα is mandatory and this might be supported via the dendritic translation of CaMKIIα.

### Role of Ca^2+^/Calmodulin-Dependent Protein Kinase Type II β

Motility and plasticity of axonal and dendritic arbors, leading to alterations in synaptic contacts (Fischer et al., 1998; Zou and Cline, 1999; Jontes et al., 2000; Colicos et al., 2001), play significant roles in developing and mature neurons. Shen et al. (1998) showed localization of CaMKIIβ to the actin cytoskeleton, thus demonstrating its role in actin-related morphology modifications. CaMKIIβ overexpression increased the number of synapses whereas inhibiting CaMKIIβ caused significant reduction in motility of filopodia as well as in small dendritic branches with long-term decrease in the degree of dendritic arborization (Fink et al., 2003). In developing hippocampal neurons, CaMKIIβ promotes arborization of the dendritic tree whereas in mature neurons, it has a strong morphogenic effect, leading to dendritic remodeling rather than overall arborization. CaMKIIβ, and not CaMKIIα is expressed in early development when the neurons build the dendritic arbor (Bayer et al., 1999). Even in the hippocampal region where CaMKIIα expression is exceedingly high, CaMKIIβ dominates during the first postnatal week, thus implying its direct role in morphogenic activity. A small insert in CaMKIIβ is responsible for its F-actin localization and for selective upregulation of dendritic motility. Wang Q. et al. reported that CaMKIIβ that has a longer linker of 93 amino acids (aa) binds more strongly and efficiently to F-actin than does CaMKIIα which has only a 30 aa linker (Wang Q. et al., 2019). They show that peptides derived from the regulatory, linker and association domains of CaMKIIβ can bind F-actin. Based on simulations, they calculated that about 20% of free energy of binding is contributed by the regulatory domain. The remaining energy is derived from the linker and association domains with nearly equal contribution. The linker domain is flexible (Myers et al., 2017) and contributes to the thermodynamics of binding unlike the association domain which has higher rigidity and thus helps in maintaining strict geometry between CaMKIIβ and the bound actin filaments. Thus, the formation of the CaMKII/F-actin complex requires the linker, regulatory and association domains of CaMKIIβ (Wang Q. et al., 2019).

When a short sequence of the variable region of CaMKIIβ was inserted in CaMKIIα, a partial colocalization and partial effect on the dendritic morphology was observed. Thus, neurons high in β isoform would have higher degree of arborization with larger numbers of synapses, an example being the cerebellar neurons having persistently high CaMKIIβ levels than in neurons in the forebrain (Miller and Kennedy, 1985). This is reflected in the highly branched morphology of cerebellar neurons when compared to neurons present in the forebrain.

Another important question is how CaMKIIβ is activated. One report suggested that actin and Ca^2+^/CaM involve in competitive binding to CaMKIIβ (Shen and Meyer, 1999). Fink et al. (2003) reported the involvement of Ca^2+^/CaM binding to CaMKII for dendritic mobility. Ca^2+^/CaM levels present in the unstimulated neurons were sufficient to induce CaMKIIβ-dependent dendritic extension/motility. Hence, Ca^2+^/CaM stimulus provided by basal neuronal activity in cultures is sufficient for the morphogenic function of CaMKIIβ. Since autophosphorylation at Thr^287^, which requires Ca^2+^/CaM binding, was possible at basal conditions (25% of CaMKII phosphorylation) (Molloy and Kennedy, 1991), sufficient Ca^2+^/CaM should be present during basal neuronal activity leading to partial CaMKIIβ activation. In contrast, CaMKIIα requires stronger stimulation to activate AMPA receptors. Thus, differential expression of the two CaMKII isoforms leads to either strengthening of the synapse if CaMKIIα function dominates or filopodia extension with synapse formation if CaMKIIβ dominates.

The mRNA of CaMKIIα, and not β is present in the dendrites and hence the protein translated in dendrites would have CaMKIIα homomers which would not be actin localized. The mixed population of both the isoforms, translated in the cell body would create α/β hetero-oligomers that might bind to actin and regulate filopodia extension and synapse formation (Mori et al., 2000; Aakalu et al., 2001).

Protein kinase C-mediated phosphorylation of CaMKIIβ is required for maintenance of spine morphology. PKC phosphorylates CaMKIIβ at Ser^315^ during group I metabotropic glutamate receptor (mGluR1) signaling which results in CaMKIIβ/F-actin complex dissociation thereby repressing formation and elongation of spines in mature Purkinje cells (Sugawara et al., 2017).

Puram et al. (2011) found a centrosomal targeting sequence (CTS) within the variable region of CaMKIIβ. The CTS mediates the required CaMKII - pericentriolar material 1 (PCM1, a centrosomal targeting protein) interaction which is required for CaMKII localization to the centrosome. In the centrosome, CaMKIIβ phosphorylates the E3 ubiquitin ligase Cdc20-APC (cell division cycle 20–anaphase promoting complex) at Ser^51^, thereby inducing Cdc20 dispersion from the centrosome and thus inhibiting centrosomal Cdc20-APC activity. This triggers the switch to retraction mode from growth of the dendrites. This CaMKIIβ function at the centrosome is independent of CaMKIIα.

### Ca^2+^/Calmodulin-Dependent Protein Kinase Type II Phosphorylation States in Spine Size and Regulation

Spine size and synaptic strength were shown to covary in experiments involving photolysis of caged glutamate, which is present in individual spines (Matsuzaki et al., 2004; Zhang et al., 2008). The spines present on dendrites can vary in size (Lisman and Harris, 1993), which might correlate with postsynaptic strength of the synapse at that particular spine (Matsuzaki et al., 2001; Asrican et al., 2007). It is known that by overexpressing autonomous (T286D)-CaMKIIα in CA1 hippocampal cells, there is enhancement in the synaptic strength with Thr^305^/Thr^306^ sites not being phosphorylated. But there is a decrease in synaptic strength when Thr^305^/Thr^306^ sites are phosphorylated (Lisman et al., 2012). Interestingly, Pi et al. (2010) showed that CaMKII and its various phosphorylation states can regulate spine size. They found that all autonomous forms of CaMKII can increase spine size. In other words, CaMKII leads to spine enlargement irrespective of Thr^305^/Thr^306^ phosphorylation. Also, the T286D/T305D/T306D form can increase spine size but at the same time decrease synaptic strength. Thus, the mechanisms through which CaMKII regulates spine structure and synaptic strength have different levels of dependence on the phosphorylation state of the enzyme. A T286D form with an additional mutation, K42R, that inhibits enzymatic activity, could actually enhance spine size, with no effect on synaptic strength, thus showing the importance of the structural (non-enzymatic) role of CaMKIIα in this postsynaptic process. Thus, the overall process might involve two steps in which initial enzymatic activity is required for initiating autophosphorylation at Thr^286^ followed by spine enlargement that does not require enzymatic activity. This explains why the kinase-dead T286D mutant (K42R/T286D) can support spine enlargement but not the T286A mutant (Pi et al., 2010).

### Role of Presynaptic Ca^2+^/Calmodulin-Dependent Protein Kinase Type II in Axon Terminal Growth

Extensive structural remodeling on the presynaptic and postsynaptic sides of the synapse is important for synaptogenesis. The axon growth cone is very dynamic as it responds to its surrounding signals ultimately growing toward the target region forming the synapse (Nesler et al., 2016). Alterations in axon terminals occur very fast and also at distant sites from the cell body. To enable these changes, the local machinery should be active and working in the growth cone and presynaptic boutons.

Ca^2+^ is an important secondary messenger in axon growth and guidance (Sutherland et al., 2014). Increased intracellular Ca^2+^ levels can activate even enzymes such as protein kinase A (PKA) through S100A1, a Ca^2+^-binding protein (Melville et al., 2017). Ca^2+^ influx results in activating Ca^2+^/CaM-dependent enzymes like calcineurin (CaN) and CaMKII (Faas et al., 2011). Activation of CaMKII and PKA promotes attraction of the growth cone toward external cues and dual inhibition of both the enzymes leads to repulsion (Wen et al., 2004). Synapsin is an important target for phosphorylation by CaMKII in the presynaptic nerve terminals. The association of synapsin with synaptic vesicles is reversible and it facilitates vesicle clustering and presynaptic plasticity. This mechanism is regulated by phosphorylation at specific sites by CaMKII and PKA (Stefani et al., 1997; Hosaka et al., 1999). Synapsin gets redistributed to sites of activity-dependent axon terminal growth and thus regulates outgrowth via a PKA-dependent pathway (Vasin et al., 2014).

CaMKII expression is post-transcriptionally regulated at the level of translation by the microRNA (miRNA) containing RNA-induced silencing complex (RISC) (Ashraf et al., 2006). Nesler et al. (2013) observed that growth of new synaptic boutons in response to spaced depolarization requires the function of activity-regulated neuronal miRNAs including miR-8, miR-289 and miR-958 in *Drosophila* larval ventral ganglia. This suggests that mRNAs encoding synaptic proteins might be regulated by these miRNAs. The fly CaMKII 3′UTR has two putative binding sites for activity-regulated miR-289 (Ashraf et al., 2006). It is also reported that miR-148a/b can target CaMKIIα through bioinformatics analysis and luciferase assay (Liu et al., 2010). In animal models of schizophrenia wherein the levels of miR-148b were significantly upregulated, increased levels of CaMKIIα transcript did not lead to a concomitant increase in protein levels (Gunasekaran et al., 2022), implying miR-148b involvement in regulation of CaMKIIα *in vivo*. Knockdown of CaMKII in the presynaptic compartment using transgenic RNAi, disrupted activity-dependent presynaptic growth as it prevented the formation of new ghost boutons in response to spaced stimulus. Abundant levels of phosphorylated CaMKII were found at the presynaptic axon terminal. Spaced stimulation leads to accumulation of a significant amount of total CaMKII protein in the axon terminals. This increase was blocked by treatment with either the translational inhibitor cycloheximide or presynaptic overexpression of miR-289 suggesting a translation-dependent mechanism. Similarly, presynaptic CaMKII has been implicated in controlling both bouton number and morphology during development of the larval neuromuscular junction (NMJ) (Nesler et al., 2016). Presynaptic CaMKII has also been shown to be involved in axon pathfinding in cultured neurons of Xenopus (Wen et al., 2004).

## Activation in Response to Voltage Gated Calcium Channels

Voltage gated calcium channels (VGCCs) are present throughout the neuronal membrane and are a major source of Ca^2+.^ especially in dendritic spines after a depolarization of the membrane. Different subtypes of VGCCs are known with distinct functions; mainly involved in Ca^2+^ influx into the cell as well as in regulating gene transcription. Activation of dendritic VGCCs can generate LTP, STP (short-term potentiation) or LTD. Perhaps because of the distinct subcellular localization of VGCCs, LTP induced due to their activation may use mechanisms distinct from NMDAR-dependent LTP (Malenka and Nicoll, 1999). With aging, LTP induction through NMDAR becomes lesser compared to VGCC-dependent LTP, as shown by the limited sensitivity of LTP generated in slices from older rats to NMDAR antagonists and increased sensitivity to antagonists of L-type VGCC (Izumi and Zorumski, 1998). Studies have also shown that repetitive activation of VGCCs is involved in LTD (Pöschel and Manahan-Vaughan, 2007) in a Ca^2+^-dependent manner. Among the various categories of VGCCs, L-type VGCCs are mainly involved in synaptic plasticity mechanisms.

In the CA1 area of hippocampus, an LTP component has been found that is dependent only on the activation of VGCCs without NMDAR (Grover and Teyler, 1990; Alkadhi, 2021) which was later termed as VDCC LTP. Ca^2+^ entry through VGCCs mediates LTP at thalamic input synapses to the lateral nucleus of amygdala, which may be mechanistically different from the NMDAR-dependent form of plasticity found in the hippocampus but is still dependent on activated CaMKII (Weisskopf et al., 1999). The conditional hippocampus/neocortex Cav1.2 (L-type VGCCs) KO mouse demonstrates an essential role of Cav1.2 in CREB signaling during LTP and spatial learning (Moosmang et al., 2005). In the cortical neurons, activation of T-type VGCCs enhanced LTP and CaMKII autophosphorylation (Moriguchi et al., 2012a). Even in the NMDAR-dependent mechanisms of LTP and LTD (Di Biase et al., 2008), Cav channels are involved (Zhao et al., 2021) by enhancing Ca^2+^ influx into the synaptic site and through CREB mediated events.

Upon aging, the expression of NMDAR diminishes and its subunit composition also changes (Zhao et al., 2009), whereas VGCCs, especially the L-type channels, increase in expression (Thibault and Landfield, 1996; Wang and Mattson, 2014) and can majorly involve in LTP or LTD mechanisms. Activation of L-type VGCCs, especially Cav1.2 localized in the postsynaptic membrane (Patriarchi et al., 2018) leads to Ca^2+^ influx into the spine, which can activate CaMKII. Even if the expression levels of GluN2B are lower, CaMKII can still tether to the postsynaptic site by binding with the C-terminus of Cav1.2 (Hudmon et al., 2005). This binding, however, does not lead to constitutively active CaMKII and hence, cannot support molecular memory. The enzyme tethered at the membrane can easily get activated with the trains of depolarization stimulus and can facilitate further Ca^2+^ influx through these channels (Ca^2+^-dependent facilitation).

## Role of Ca^2+^/Calmodulin-Dependent Protein Kinase Type II in Long Term Depression

**FIGURE 4:**
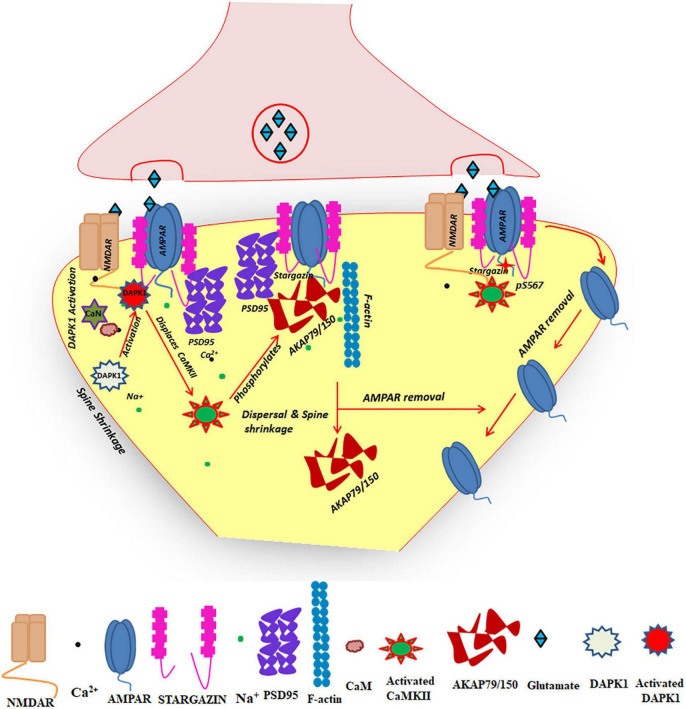
The schematic representation showing the role of CaMKII in LTD. The low tetanic stimulation leading to LTD activates more phosphatases than kinases. Calcineurin thus activated can activate DAPK1 and it can translocate to GluN2B where CaMKII binds. The activation of DAPK1 can even displace activated CaMKII generated under minimal Ca^2+^ stimulus from its binding with GluN2B. The role of CaMKII in LTD involves inhibition of AMPAfication and facilitation of spine shrinkage. Phosphorylation of GluA1 of AMPAR at Ser^567^ obstructs AMPAfication of synapses; CaMKII mediated phosphorylation and depalmitoylation of AKAP79/150 results in its synaptic elimination. Since AKAP79/150 is a major adapter for many proteins required for LTP, its elimination due to dissociation from F-actin can result in AMPAR endocytosis and spine shrinkage.

LTD is an activity-dependent reduction in the efficacy of neuronal synapses (Malenka and Nicoll, 1999) and is thought to be involved in learning and memory. It brings about a long-lasting decrease in synaptic strength or a reversal of LTP mechanisms. LTD is triggered by synaptic activation of either NMDARs or metabotropic glutamate receptors (mGluRs). A low frequency stimulation (LFS) of NMDARs (700–900 pulses at 1 Hz) can activate LTD mechanisms (Figure 4). If the Ca^2+^ influx is low in intensity (if the activation is only for a postsynaptic compartment), it will majorly activate phosphatases and result in LTD (Baltaci et al., 2019). Initially it was thought that protein kinases are required for LTP and phosphatases are involved in LTD. But recent findings suggest that kinases are involved in LTD mechanisms also. It has been noted that the bath application of CaMKII inhibitor KN-62 could block LTD during low-frequency SC collateral stimulation (1 Hz/15 min) (Stanton and Gage, 1996). Experiments with CaMKIIα KO mice also pointed to the role of CaMKII in LTD (Stevens et al., 1994). Even though these initial experiments indicated the role of CaMKII in LTD, the exact mechanism by which CaMKII participates in the process is unknown. In contrast to the previously accepted dogma, it has also been shown by using T286A mutant mouse that Thr^286^ autophosphorylation is a requisite for LTD (Coultrap et al., 2014). The most recent studies on CaMKII autophosphorylation indicates that the autophosphorylation at Thr^305/306^ is selectively induced by LTD stimuli and the mutation of these residues impairs LFS-induced LTD but not HFS-induced LTP (Cook et al., 2021). Both the autophosphorylations are necessary for LTD but the exact role of Thr^286^ with respect to Thr^305/306^ in LTD remains controversial. The death-associated protein kinase 1 (DAPK1) can regulate CaMKII-GluN2B interaction to facilitate LTD. DAPK1 is a CaM kinase family member and is enriched in excitatory synapses. They can bind to GluN2B at a site overlapping the CaMKII binding site. The enzyme gets activated by CaN, a Ca^2+^-activated protein phosphatase. LTD-stimuli can activate DAPK1 in hippocampal slices in a CaN-dependent manner. Inhibition of DAPK1 or CaN allowed the accumulation of CaMKII at excitatory synapses after LTD-stimuli (Goodell et al., 2017). This indicates that during LTD, DAPKI activated by phosphatases will compete for GluN2B binding and would reduce the binding of activated CaMKII generated by the low frequency stimuli.

CaMKII can phosphorylate Ser^567^ residue of GluA1 subunit of AMPAR, a unique phosphorylation site for CaMKII in the C-terminal loop of GluA1. The C-terminal tail of GluA1 is involved in AMPAR trafficking from extra-synaptic pool to the synapses. Phosphorylation of GluA1 at Ser^567^ by CaMKII inhibits AMPAR trafficking to the synapses (Lu et al., 2010). It has been noted that LTD-inducing stimulation of hippocampal slices produced a robust phosphorylation of Ser^567^ whereas LTP-inducing stimulus could yield only Ser^831^ phosphorylation. The differential phosphorylation of GluA1 by CaMKII under the two synaptic plasticity conditions underlies the role of CaMKII in LTD (Coultrap et al., 2014).

In contrast to spine enlargement in LTP, LTD is associated with spine shrinkage aided by the removal of the AMPA receptor regulatory scaffold protein, A-kinase anchoring protein (AKAP) 79/150. The synaptic removal of AKAP79/150 is brought about by the phosphorylation of the substrate sites within the AKAP79/150 N-terminal polybasic membrane-cytoskeletal targeting domain (residues 1–153) by CaMKII. Phosphorylation by CaMKII inhibits AKAP79/150 association with F-actin, thus facilitating AKAP79/150 removal from spines (Figure 4). In addition to the direct phosphorylation of AKAP79/150, CaMKII is also responsible for its depalmitoylation on two Cys residues within the N-terminal targeting domain. Depalmitoylation also promotes synaptic elimination of AKAP79/150. Since the protein harbors PKA and protein phosphatase 2B (PP2B) at the PSD, it can regulate both synaptic insertion and elimination of AMPARs. Under LTP stimulation, PKA can phosphorylate Ser^845^ of GluA1 of AMPAR and thereby more AMPAR trafficking to the synapse occurs, whereas in LTD conditions due to the elimination of AKAP79/150 along with activation of phosphatases, AMPAR dephosphorylation at Ser^845^ and its endocytosis is promoted which eventually leads to spine shrinkage (Woolfrey et al., 2018).

The stimulation pattern-dependent activation of NMDAR that yields either LTP or LTD, causes activation of CaMKII in either case. With the differing stimuli the enzyme targets different substrates and thereby activates specific signaling mechanisms to yield either form of synaptic plasticity.

## Ca^2+^/Calmodulin-Dependent Protein Kinase Type II in Signaling Complexes in Glutamatergic Synapses

CaMKII plays an important role in several physiological pathways including synaptic plasticity and hence its localization in the cytosol and PSD are crucial determinants of its function. Immunoelectron microscopy studies show that CaMKIIα is significantly higher in dendritic shafts when compared to dendritic spines. When it gets any proper stimulus, it will abundantly translocate to the spines (Shen and Meyer, 1999; Shen et al., 2000; Ding et al., 2013). In the basal condition, more CaMKII will be available in the dendritic shaft than in spines. Whenever activation happens the activated CaMKII can translocate to the spines.

Translocated CaMKII can bind with various protein ligands in the PSD as indicated in Table 1. One such protein is densin-180, which is a core protein in the PSD that does not span the membrane. Though densin-180 is the only documented interaction partner for the association domain of CaMKII, it will not bind with CaMKII holoenzymes which contain β isoform (Penny and Gold, 2018). The PDZ domain of densin-180 contributes to its binding to α-actinin. A distinct domain of α-actinin interacts with the kinase domains of both α and β subunits of CaMKII. Thus, these three proteins can form a ternary complex in the PSD stabilized by multiple interactions (Walikonis et al., 2001). This ternary complex within the PSD is an additional mode of localization of CaMKII to PSD apart from its binding to GluN2B.

SAP97, a member of membrane-associated guanylate kinase protein family, has been implicated in the processes of targeting ionotropic glutamate receptors such as NMDARs and AMPARs at postsynaptic sites and is enriched in PSD. SAP97 shares its interaction with AKAP79/150 in addition to the C-terminal region of GluA1. AKAP79/150 in turn harbors PKC, PKA and PP2B. This molecular arrangement inside the PSD works in accordance with the stimuli received. The most important function of this complex is the regulation of AMPARs in synapses including both potentiation and trafficking. CaMKIIα displays a high degree of co-localization with SAP97. CaMKII phosphorylation of Ser^39^ in the N-terminus of SAP97 modulates trafficking of SAP97 (Mauceri et al., 2004) and the associated proteins; in contrast, CaMKII phosphorylation of Ser^232^ in the first PDZ domain of SAP97 may modulate binding of other proteins, such as NMDAR and AMPAR subunits (Nikandrova et al., 2010), especially GluA1 of AMPAR. SAP97 is in close association with AKAP 79/150, but the phosphorylation of SAP97 at Ser^39^ by CaMKII disengages AKAP79/150 from regulating GluA1-AMPARs.

Another complex associated with CaMKII in the PSD is the complex formed by SynGAP, MUPP1 and CaMKII. SynGAP and CaMKII are brought together by direct physical interaction with the PDZ domains of MUPP1, a multi-PDZ domain-containing protein (Krapivinsky et al., 2004). In this complex, SynGAP is phosphorylated by CaMKII which enhances its Ras GTPase activity which in turn promotes AMPAR trafficking as shown in Figure 3.

CaMKII has an important role in dendritic spine remodeling upon synaptic stimulation. Electron micrographic studies showed that at physiological molar ratios, single CaMKII holoenzymes cross-linked multiple F-actin filaments at random, whereas at higher CaMKII/F-actin ratios, filaments bundled. From this bundled state CaMKII is released upon Ca^2+^/CaM activation, triggering network disassembly and expansion leading to spine enlargement. Upon subsequent disappearance of Ca^2+^, compaction will occur (Khan et al., 2019).

## Role of Ca^2+^/Calmodulin-Dependent Protein Kinase Type II in Calcium Overload-Induced Excitotoxicity

Excitotoxicity is a pathological condition triggered by excessive stimulation of receptors by excitatory neurotransmitters, primarily glutamate, causing Ca^2+^ overload in the cytosol and thereby resulting in neuronal dysfunction and cell death. Increased Ca^2+^ influx and high intracellular Ca^2+^ ([Ca^2+^]_i_) rise trigger gene expression (Ortuño-Sahagún et al., 2012) and long-lasting activation of CaMKIIα in hippocampal neurons (Otmakhov et al., 2015). Autophosphorylation of CaMKII at Thr^253^, Thr^286^ (Vest et al., 2010; Otmakhov et al., 2015; Rostas et al., 2017) and simultaneous S-nitrosylation at Cys^280^/Cys^289^ by nitric oxide (NO) (Coultrap and Bayer, 2014) generates autonomous activity of the kinase during excitotoxic cell death. Activated CaMKII redistributes to the spines (Otmakhov et al., 2015), promotes its interaction with synaptic GluN2B (Wang N. et al., 2014; Buonarati et al., 2020) and mediates the NMDA-induced caspase-3-dependent cell death pathway (Goebel, 2009). During a glutamate-induced excitotoxic event, CaMKII can also modulate the activity of neuronal nitric oxide synthase (nNOS) (Araki et al., 2020), can cause axonal degeneration via necroptosis (Hou et al., 2009; Arrazola and Court, 2019) and also contribute to the regulated necrosis (RN) pathway (Wang S. et al., 2019).

Contrastingly both overexpression (Vest et al., 2010) and sustained CaMKII inhibition during excitotoxicity can exacerbate cell death of cultured neurons (Ashpole and Hudmon, 2011; Ashpole et al., 2012). Loss of CaMKII activity in astrocytes results in dysregulated Ca^2+^ homeostasis and reduced glutamate uptake (Ashpole et al., 2013) by excitatory amino acid transporter 1 (EAAT1) (Chawla et al., 2017). On the whole, dysregulated CaMKII function upon excitotoxic insult shifts the tight homeostatic balance maintained between kinases and phosphatases in the cell, resulting in dysfunction of excitatory synaptic transmission (Farinelli et al., 2012). The following section reviews the role of CaMKII at glutamatergic synapses in a few diseases in which excitotoxicity is one of the causes.

### Alzheimer’s Disease

Alzheimer’s disease (AD) is a progressive neurodegenerative condition characterized by loss of memory and cognitive function. The presence of amyloid β (Aβ) plaques and neurofibrillary tangles (NFTs) composed of hyperphosphorylated tau protein, is the distinctive feature in AD neuropathology. CaMKII catalyzes the hyperphosphorylation of tau protein at multiple Ser/Thr sites in the AD brain (Yoshimura et al., 2003). Loss of synapses and cognitive decline associated with AD positively correlate to the accumulation of soluble Aβ (Lue et al., 1999; Näslund et al., 2000; Almeida et al., 2005), which leads to reduced CaMKII activation (Zeng et al., 2010; Ly and Song, 2011; Ghosh and Giese, 2015) and inhibition of LTP-induced CaMKII trafficking to excitatory synapses (Cook et al., 2019). A significant reduction in the density and number of synapses (Terry et al., 1991; Scheff and Price, 1993, 1998; Scheff et al., 2006) and altered expression of synaptic proteins (Masliah et al., 2001; Almeida et al., 2005) contributes to synaptic dysfunction and cognitive decline in the AD brain.

In amyloid precursor protein (APP) transgenic mice, Aβ-induced change in CaMKII subcellular distribution aids in the removal of AMPARs from the synaptic membrane (Gu et al., 2009). Opazo et al. (2018) showed that oligomeric forms of Aβ peptide engage in synaptic metaplasticity via aberrant activation of CaMKII, mediated through GluN2B-containing NMDARs, which leads to LTP deficits and destabilization of AMPARs in the early stages of AD.

### Epilepsy

Epilepsy is a neurological disorder characterized by recurrent seizures, caused by abnormal brain activity. A strong epileptic stimulus can induce alterations in the composition of PSD proteins (Wyneken et al., 2001) and loss of CA3 cells in a kainic acid (KA)-induced seizure model, wherein hippocampal injury correlates with increased CaMKII activity (Lee et al., 2001). Activation of CaMKIIα is concomitant with a reduction in density of hippocampal dendritic spines and spine PSDs during epileptiform activity (Zha et al., 2009). Also, CaMKII activation via L-type VGCCs and NMDARs are essential for the development and maintenance of an *in vitro* kindling-like state and EPSP-spike potentiation in CA1 pyramidal cells (Semyanov and Godukhin, 2001).

However, a few studies have reported an NMDAR-dependent reduction in CaMKII activity with increased neuronal excitability (Kochan et al., 1999; Churn et al., 2000). Regulation of CaMKII activity during seizures either by the reversible formation of inactivated CaMKII (Yamagata and Obata, 2004; Yamagata et al., 2006) or by modulating different CaMKII isoforms (Murray et al., 2003; Savina et al., 2013), can prevent excessive CaMKII activation due to Ca^2+^ overload (Yamagata et al., 2006). Recently, Vieira et al. (2020) functionally characterized the epilepsy-associated *de novo* variant of GluN2A, S1459G. This mutation disrupts CaMKIIα phosphorylation of GluN2A resulting in defects in NMDAR trafficking and reduced synaptic function (Vieira et al., 2020).

### Huntington’s Disease

Huntington’s disease (HD) is an autosomal, dominantly inherited disorder caused by the expansion of a polyglutamine repeat in the N-terminus of the huntingtin (htt) protein. Progressive and selective degeneration of the striatal medium spiny neurons (MSNs) in HD results in abnormalities of movement, cognition, personality and mood. Being an abundant protein in striatal MSNs (Erondu and Kennedy, 1985), reduced levels of both CaMKII and CaMKII-Thr^286^ phosphorylation have been reported in various mouse models of HD (Deckel et al., 2001, 2002a,b; Brito et al., 2014; Blum et al., 2015; Gratuze et al., 2015). Altered expression levels of CaMKII in the hippocampus can disrupt GluA1-Ser^831^ phosphorylation (Brito et al., 2014) and disturb AMPAR surface diffusion (Zhang et al., 2018). CaMKII inhibition in striatal MSNs causes a reduction in functional glutamatergic synapses and an enhancement in intrinsic excitability (Klug et al., 2012). Although the role of altered CaMKII function in HD is not extensively studied, it is evident that it could contribute to cognitive dysfunction observed in HD (Giralt et al., 2012; Zhang et al., 2018).

### Parkinson’s Disease (PD)

Parkinson’s disease (PD) is a progressive neurodegenerative movement disorder caused by degeneration of dopaminergic neurons in the substantia nigra, that project to the striatum. At the molecular level, dopamine (DA) can modulate or gate the cortical glutamatergic inputs onto striatal MSNs (Freund et al., 1984; Gardoni and Bellone, 2015). Striatal DA depletion causes selective loss of dendritic spines and glutamatergic synapses on striatopallidal MSNs (Day et al., 2006) and differentially affects the expression and phosphorylation of glutamate receptor subunits and CaMKIIα (Brown et al., 2005; Gardoni et al., 2010; Koutsokera et al., 2014).

Dopamine denervation *in vivo* induces an increase in CaMKIIα-Thr^286^ phosphorylation in the striatum (Brown et al., 2005; Koutsokera et al., 2014), concurrent with increased recruitment of activated CaMKIIα to GluN2A-GluN2B subunits (Picconi et al., 2004). On the other hand, reduced levels of CaMKIIα autophosphorylation and GluA1-Ser^831^ phosphorylation in the hippocampus correlates with impaired CA1 LTP in 1-methyl-4-phenyl-1,2,3,6-tetrahydropyridine (MPTP)-treated mice (Moriguchi et al., 2012b). Overall, DA deficiency can induce deficits in synaptic plasticity and motor behavior by altering striatal glutamatergic signaling and CaMKII activity (Picconi et al., 2004; Brown et al., 2005; Deutch, 2006; Paillé et al., 2010; Moriguchi et al., 2012b; Koutsokera et al., 2014).

### Cerebral Ischemia

Cerebral ischemia is a condition in which restricted blood supply to the brain causes tissue damage and cell death. Excess glutamate release and high [Ca^2+^]_i_ trigger a range of downstream neurotoxic cascades leading to apoptosis or necrosis (Szydlowska and Tymianski, 2010). Ca^2+^ influx ensuing an ischemic insult significantly increases NMDAR-mediated activation of CaMKII (Meng et al., 2003) followed by its phosphorylation at Thr^253^ (Gurd et al., 2008) and Thr^286^ (Shamloo et al., 2000; Matsumoto et al., 2002). CaMKII-Thr^253^ autophosphorylation enhances its association with PSD (Migues et al., 2006) and induces the persistent activation of the enzyme (Rostas et al., 2017). Oxidation of Met^281/282^ (Cys^281^/Met^282^ in CaMKIIα) in the auto-regulatory domain of the enzyme, by reactive oxygen species (ROS) generated during glutamate excitotoxicity and oxidative stress, can also lead to autonomous activity of the kinase (Anderson, 2015), which in turn augments reperfusion injury in acute ischemic stroke (Gu et al., 2016; Qu et al., 2019; Zhang et al., 2021). Autophosphorylated CaMKII translocates to the synaptic membrane (Matsumoto et al., 2004), binds to synaptic GluN2B (Buonarati et al., 2020) and phosphorylates serine residue(s) of the GluN2B subunit (Meng and Zhang, 2002; Meng et al., 2003) to mediate ischemic cell death. However, a recent study by Tullis et al. (2021), reported that neuronal death in global cerebral ischemia *in vivo* is promoted by the binding of CaMKII to GluN2B and not by CaMKII-mediated GluN2B-Ser^1303^ phosphorylation (Kumar et al., 2019; Buonarati et al., 2020; Tullis et al., 2021). CaMKII activation dependent on NMDARs or L-type VGCCs can also phosphorylate serine residues of GluR6 subunit of kainate receptors via the assembly of GluR6-PSD95-CaMKII signaling module in cerebral ischemia injury (Hao et al., 2005; Xu et al., 2010).

The changes observed in expression levels and activity of CaMKII are dependent on the duration of ischemic insult (Gurd et al., 2008), which in turn can regulate NMDAR-mediated field excitatory postsynaptic potentials (fEPSPs) (Wang N. et al., 2014). Likewise, 10 min oxygen-glucose deprivation (OGD) treatment *in vitro* can induce NMDAR-mediated postischemic LTP, mediated by CaMKII-NMDAR interaction and NMDAR trafficking to the membrane (Wang N. et al., 2014).

### Traumatic Brain Injury

Traumatic brain injury (TBI) is a disruption in the normal function of the brain caused by an external mechanical force. It is associated with the release of excitatory amino acids, particularly glutamate, in the extracellular space (Faden et al., 1989; Chamoun et al., 2010). Overactivation of glutamate receptors (Faden et al., 1989; Liu et al., 2017) and elevated levels of [Ca^2+^]_i_ (Deshpande et al., 2008; Sun et al., 2008) transiently activates CaMKIIα (Atkins et al., 2006; Folkerts et al., 2007; Liu et al., 2017) and CaMKIIδ (Zhang et al., 2012). Alterations in NMDAR function, CaMKIIα expression and dendritic spine anatomy in the hippocampus prevent LTP induction after lateral fluid percussion injury (Schwarzbach et al., 2006), thereby causing cognitive impairment often associated with CNS trauma (Atkins et al., 2006; Schwarzbach et al., 2006; Folkerts et al., 2007; Deshpande et al., 2008). Long-term alterations in Ca^2+^ homeostasis mechanisms (Sun et al., 2008) contributes to morbidity and mortality following TBI.

## Functional Implications of Ca^2+^/Calmodulin-Dependent Protein Kinase Type II Mutations in Synaptic Plasticity

CaMKII plays a versatile role in different regulatory processes involved in synaptic plasticity. This section reviews the different CaMKII mutant animal models generated to study the physiological role of the kinase in synaptic plasticity and its associated behavioral phenotype. Targeted disruption of CaMKIIα/β/γ function *in vivo* dysregulates different types of synaptic plasticity (Silva et al., 1992a; Stevens et al., 1994; Mayford et al., 1995; Giese et al., 1998; Elgersma et al., 2002; Miller et al., 2002; Cho et al., 2007; van Woerden et al., 2009; Yamagata et al., 2009; Yin et al., 2017; Cohen et al., 2018; Kool et al., 2019) and impairs learning (Silva et al., 1992b,^1996^; Bach et al., 1995; Giese et al., 1998; Elgersma et al., 2002; Irvine et al., 2005; Yamagata et al., 2009; Borgesius et al., 2011; Achterberg et al., 2014; Cohen et al., 2018), memory (Miller et al., 2002; von Hertzen and Giese, 2005; Cho et al., 2007) and the emotional state (Chen et al., 1994; Yamasaki et al., 2008; Hasegawa et al., 2009; Bachstetter et al., 2014). Although the behavior exhibited varies slightly with the genetic background of the mouse strain used (Gordon et al., 1996; Silva et al., 1996; Hinds et al., 1998; Need and Giese, 2003), the molecular and electrophysiological alterations remain largely unchanged.

### Ca^2+^/Calmodulin-Dependent Protein Kinase Type II α

#### Ca^2+^/Calmodulin-Dependent Protein Kinase Type II α Global Knockout Mice

Silva et al. (1992a) reported the production of the first genetically altered mice lacking the α subunit of CaMKII. LTP, STP and LTD were either absent or significantly attenuated in the sensory neocortex and hippocampal slices from young homozygous CaMKIIα^–/–^ KO mice (Silva et al., 1992a; Stevens et al., 1994; Kirkwood et al., 1997; Hinds et al., 1998; Elgersma et al., 2002). Long-term plasticity and reversal of LTP were normal in the CA1 hippocampal region of heterozygous CaMKIIα^+/–^ mice (Silva et al., 1996; Elgersma et al., 2002); however, they exhibited impaired short-lived plasticity (SLP) and paired-pulse facilitation (PPF) and an enhanced post-tetanic potentiation (PTP) response expressed within seconds of stimulation (Silva et al., 1992a,^1996^; Chapman et al., 1995; Hojjati et al., 2007).

Plasticity deficits due to either partial or complete loss of CaMKIIα activity manifest as abnormalities in various behavioral paradigms. CaMKIIα null mutant mice have been reported to exhibit pronounced deficits in spatial learning (Silva et al., 1992b; Elgersma et al., 2002; Achterberg et al., 2014), working memory (Yamasaki et al., 2008) and Pavlovian fear conditioning (Chen et al., 1994; Silva et al., 1996; Elgersma et al., 2002; Achterberg et al., 2014). Dysregulated emotional states like increased aggression, decreased anxiety and depression-like behavior and an exaggerated infradian rhythm have also been observed in CaMKIIα^+/–^ mice (Silva et al., 1992b; Chen et al., 1994; Yamasaki et al., 2008).

Dysfunction of the dentate gyrus (DG) due to the immaturity of DG neurons (Yamasaki et al., 2008; Matsuo et al., 2009) and ectopic projection of mossy fibers (Nakahara et al., 2015), causes suppressed induction of activity-dependent genes like *c-fos* and *arc*, resulting in altered behavior exhibited by CaMKIIα KO mice (Yamasaki et al., 2008; Matsuo et al., 2009). Disrupted regulation of *Zif268* gene expression and growth associated protein 43 (GAP43), a synaptogenesis marker, by CaMKIIα^+/–^ mutation can also impair the maturation of cortical circuits necessary for remote memory (Frankland et al., 2004).

#### Ca^2+^/Calmodulin-Dependent Protein Kinase Type II α-Thr^286^ Mutant Mice (T286A/T286D)

The Ca^2+^/CaM-independent, autonomous state of CaMKIIα, induced by autophosphorylation of Thr^286^, is required for NMDAR-dependent LTP and LTD at CA1 pyramidal cells (Giese et al., 1998), spatial learning (Giese et al., 1998; Need and Giese, 2003), fear learning (Irvine et al., 2005, 2011) and regulation of synapse development *in vivo* (Gustin et al., 2011). During induction of synaptic plasticity, CaMKIIα-Thr^286^ phosphorylation is essential for optimal integration of Ca^2+^ signals; however, it is dispensable for LTP maintenance and memory (Irvine et al., 2005; Chang et al., 2017). High-frequency synaptic stimulation can rescue impaired LTP induction in CA1 neurons from *Camk2a*^T286A^ mice (Chang et al., 2017). Although L-LTP could not be induced at CA1 synapses of T286A mutants (Irvine et al., 2011), mTOR-mediated upregulation of PSD95 expression and a persistent generation of multi-innervated spines (MIS) can contribute to LTM formation in these mutant animals where functional strengthening of synapses is impaired (Radwanska et al., 2011).

The deficit in spatial learning of CaMKIIα-T286A mutant mice is due to decreased spatial selectivity, stability and experience-dependent tuning of CA1 hippocampal place cells (Cho et al., 1998; Cacucci et al., 2007) and an impaired precision of spatial memory (Śliwińska et al., 2020). Pre-adolescent KI mice had disruption in synaptic targeting of CaMKII and enhanced activity of GluN2B-containing-NMDARs at CA3-CA1 synapses along with impaired cognition and anxiety phenotypes (Gustin et al., 2011). The T286A knockin (KI) mutants have normal neurogenesis in their DG (Kee et al., 2007). Therefore, alternate signaling mechanisms involving either PKA or CaMKIIβ are activated in the absence of CaMKIIα autophosphorylation at excitatory synapses in the neonatal rodent hippocampus (Yasuda et al., 2003), hippocampal inhibitory interneurons (Lamsa et al., 2007) and the medial perforant path-granule cell synapses in adult mice (Cooke et al., 2006) to induce LTP.

Constitutive expression of the Ca^2+^-independent, autonomously active form of CaMKIIα (CaMKIIα-T286D) *in vivo* favors LTD at LTP-inducing θ frequencies (5–10 Hz) and consequently influences spatial learning and fear conditioning (Bach et al., 1995; Mayford et al., 1995, 1996; Wiedenmayer et al., 2000; Bejar et al., 2002; Yasuda and Mayford, 2006). The use of the tetracycline transactivator (tTA) system to limit the expression of CaMKIIα-T286D regionally and temporally, has shed light on the role of CaMKIIα signaling in synaptic plasticity during development, memory encoding and memory storage (Mayford et al., 1996; Glazewski et al., 2001; Bejar et al., 2002; Yasuda and Mayford, 2006). CA1 hippocampal place cells in these mutant animals are less common, less precise and less stable, thereby affecting spatial memory storage (Rotenberg et al., 1996).

#### Ca^2+^/Calmodulin-Dependent Protein Kinase Type II α-Thr^305^/Thr^306^ Mutant Mice

Inhibitory phosphorylation of CaMKIIα at Thr^305^/Thr^306^ is essential to modulate the association of the kinase with PSD, the threshold for induction of NMDAR-dependent LTP at SC-CA1 synapses, hippocampal-dependent spatial learning and fear conditioning, reversal learning and to induce LTP at inhibitory synapses (iLTP) (Elgersma et al., 2002; Cook et al., 2021). Phosphorylation of CaMKIIα-Thr^305^/Thr^306^ during an excitatory LTD stimulus blocks the translocation of CaMKIIα to glutamatergic excitatory synapses and directs CaMKIIα to GABAergic inhibitory synapses to induce iLTP. In this way, Thr^305^/Thr^306^ phosphorylation governs the fundamental LTP vs. LTD decision at excitatory synapses (Cook et al., 2021). Similar to CaMKIIα-T286D mutant, CaMKIIα-T305D favors LTD over LTP at weak tetanic stimulations (Elgersma et al., 2002).

#### Ca^2+^/Calmodulin-Dependent Protein Kinase Type II α-K42R Mutant Mice

Similar to the CaMKIIα mutant models reviewed above, the kinase-dead CaMKIIα (CaMKIIα-K42R) KI mouse also exhibited deficits in NMDAR-dependent LTP and hippocampus-dependent learning and memory (Yamagata et al., 2009, 2018). Although the levels of PSD associated CaMKIIα and activity-dependent postsynaptic translocation of CaMKIIα were intact in the mutants, the stimulus-induced increase in spine volume was severely impaired compared to WT mice (Yamagata et al., 2009). Amygdala-dependent fear memory is only partially affected by the loss of kinase activity (Yamagata et al., 2018). Stronger conditioning or multi-trial training could achieve slight or no improvement in the memory deficits of CaMKIIα-K42R mutant mice (Yamagata et al., 2009, 2018).

#### Conditional Mutant Models

Apart from the models described above, there are a few other transgenic (Tg) mouse models generated to study specific functions of CaMKII in synaptic plasticity. The CaMKIIα-3′UTR mutant has reduced expression of the kinase in the dendrites and its association to PSD (Miller et al., 2002), with no substantial alteration in other protein constituents of the synaptic membrane (Li et al., 2007). Disruption in the local translation of the protein causes a reduction in L-LTP, memory consolidation and LTM storage, with no change in E-LTP and STM formation (Miller et al., 2002).

Using an inducible and forebrain specific CaMKIIα-F89G Tg mouse model, Joe Z. Tsien and group have shown that the levels of CaMKIIα protein can affect the degree and direction of synaptic plasticity (Wang et al., 2003, 2008). A switch between the normal and higher activity state of CaMKIIα during the memory consolidation phase can severely disrupt LTM formation. The synaptic consolidation of LTMs requires the reactivation of CaMKIIα, during the first week after training, to the level present at the time of initial learning (Wang et al., 2003); on the other hand, a shift in CaMKIIα activation status within the immediate post-learning 10 min can alter STM formation (Wang et al., 2008).

In the study reported by Achterberg et al. (2014), conditional *Camk2a* mutant mice models were employed to achieve regional and temporal specific deletion of CaMKIIα. Telencephalon-specific deletion of the *Camk2a* gene (*Camk2a^flox/Emx–Cre^*) resulted in severe deficits in spatial and contextual learning and hippocampal LTP in adult mice, whereas mice with deletion specific to Purkinje cells in the cerebellum (*Camk2a*^*flox/L*7–cre^) learned normally (Achterberg et al., 2014).

At hippocampal synapses, CaMKIIα functions non-enzymatically by limiting the size of docked vesicles (Hojjati et al., 2007) and by regulating neurotransmitter release at glutamatergic synapses (Chapman et al., 1995; Hinds et al., 2003), thereby modulating short-term presynaptic plasticity. A few of the CaMKIIα Tg mice also exhibited seizures (Butler et al., 1995; Mayford et al., 1995; Elgersma et al., 2002; Yamagata et al., 2009). With a potential role for CaMKIIα in controlling the state of emotion, these models can also be exploited in the study of neuropsychiatric diseases (Yamasaki et al., 2008; Hasegawa et al., 2009; Matsuo et al., 2009; Nakahara et al., 2015; Yamagata et al., 2018).

### Ca^2+^/Calmodulin-Dependent Protein Kinase Type II β

The first Tg mouse model of CaMKIIβ was generated by Cho et al. (2007), by selectively overexpressing CaMKIIβ-F90G in the DG. Elevated CaMKIIβ activity does not affect baseline glutamatergic neurotransmission but causes deficits in LTP (Cho et al., 2007) and in NMDAR-dependent LTD (Yin et al., 2017). The Tg mice displayed normal acquisition, retention and recall of 1-day-old LTM, but showed severe impairments in 10-day-old contextual fear memory (Cho et al., 2007) and behavioral flexibility (Yin et al., 2017). Overexpression of CaMKIIβ decreases the activity of PP1/protein phosphatase 2A (PP2A) and glycogen synthase kinase 3β (GSK3β), which can shift the direction of synaptic plasticity toward potentiation during LTD induction. This disrupts the regulation of synaptic stargazin and interrupts the internalization of AMPAR and dephosphorylation of Ser^831^ and Ser^845^ of GluA1 during NMDAR-LTD (Yin et al., 2017).

Global KO models of CaMKIIβ (*Camk2b*^–/–^) have been generated by deletion of exon sequences of the *Camk2b* gene (van Woerden et al., 2009; Bachstetter et al., 2014; Kool et al., 2016). *Camk2b*^–/–^ mice exhibited cerebellar ataxia and severe deficits in locomotion (Kool et al., 2016), motor coordination (van Woerden et al., 2009), balance and cognition (Bachstetter et al., 2014). Interestingly, they showed reduced anxiety in a gene dose-dependent manner (Bachstetter et al., 2014).

Loss of CaMKIIβ, in *Camk2b*^–/–^ mice, results in bidirectional inversion of postsynaptic plasticity at the parallel fiber (PF)-Purkinje cell (PC) synapse (van Woerden et al., 2009; Pinto et al., 2020). Failure of proper targeting of CaMKIIα to dendritic spines in the absence of CaMKIIβ in the *Camk2b*^–/–^ mice results in impaired hippocampal NMDAR-dependent LTP and fear learning (Borgesius et al., 2011). This disrupted phenotype was absent in the *Camk2b*^A303R/A303R^ KI model in which Ca^2+^/CaM-dependent kinase activation of CaMKIIβ is disabled but F-actin binding and bundling functions are preserved (Borgesius et al., 2011). During LTP induction, a transient detachment of CaMKIIβ from F-actin, triggered by Ca^2+^ influx through glutamate receptors and the associated autophosphorylation of the F-actin binding region, is necessary for spine enlargement and LTP maintenance (Kim et al., 2015). Persistent binding of CaMKIIβ to F-actin in the amygdala could be causing deficits in LTP (Kim et al., 2015, 2019). To study the regulation of CaMKIIβ-F-actin interaction by autophosphorylation, a KI mouse model was generated by substituting Thr and Ser residues with Ala at exon 13 of *Camk2b* (CaMKIIβ^*exon*13:TS/A^). This KI mouse exhibited reduced freezing in fear conditioning tests (Kim et al., 2019). The absence of impairment in fear learning in the CaM-binding deficient mutant reported by Borgesius et al. (2011) might be due to phosphorylation of the F-actin binding domain in the non-activable CaMKIIβ-A303R mutant by neighboring α-subunits of the same oligomer (Kim et al., 2019).

Regardless of normal hippocampal plasticity, *Camk2b*^A303R/A303R^ mice exhibited severe deficits in motor behavior. However, the autophosphorylation deficient *Camk2b* mice, *Camk2b*^T287A/T287A^, showed no significant change in locomotion compared to WT littermates, indicating a crucial role for Ca^2+^/CaM-dependent activity, but not autonomous activity in normal mouse locomotion (Kool et al., 2016). Among the different *Camk2b* conditional mutants generated (Kool et al., 2016), *Camk2b^f/f^*;*L7-cre* mice with specific loss of CaMKIIβ in cerebellar Purkinje cells showed impaired motor learning when tested for five consecutive days, indicating that cerebellar CaMKIIβ is essential for motor function (Kool et al., 2016).

### *Camk2a-Camk2b* Double Mutants

The use of single mutants of *Camk2a* or *Camk2b* to study their function during development and in the mature brain can be inadequate when crucial functions are masked by compensation by the non-deleted form. For this purpose, double mutants of both isoforms (*Camk2a*^–/–^;*Camk2b*^–/–^) were generated (Kool et al., 2019). Germline or adult deletion of both CaMKIIα and CaMKIIβ in mice is lethal. Similarly, the Ca^2+^-dependent and -independent activities of CaMKIIα and CaMKIIβ are also essential for survival. Acute deletion of both CaMKII isoforms does not overtly affect the biochemical composition of PSD. Adult loss of CaMKIIα and CaMKIIβ also abolished LTP in the hippocampal CA3-CA1 SC pathway. This deficit was absent in mice containing a specific deletion of CaMKII isoforms in the CA3 region of the hippocampus (*Camk2a*^f/f^*;Camk2b*^f/f^*;CA3-Cre*), indicating that presynaptic CaMKIIα and CaMKIIβ are dispensable for LTP at the CA3-CA1 synapses. However, deletion of CaMKII in the CA3 region resulted in significant reduction in LTP at the associational/commissural pathway (CA3-CA3 synapse) (Kool et al., 2019).

### Ca^2+^/Calmodulin-Dependent Protein Kinase Type II γ

Similar to CaMKIIα and CaMKIIβ, global CaMKIIγ KO mice (CaMKIIγ^–/–^) displayed pronounced impairments in hippocampal-dependent memory tasks and avoidance behavior (Cohen et al., 2018). Training-induced increase in the expression of plasticity genes – *BDNF*, *c-Fos* and *Arc* – was prevented in CaMKIIγ^–/–^ mice. While E-LTP was intact, L-LTP was strongly affected at SC-CA1 synapses of CaMKIIγ^–/–^ mice, indicating deficits in LTM, but not STM. KO mice harboring a selective deletion of CaMKIIγ in excitatory neurons (CaMKIIγ-exc-KO), also exhibited impaired spatial learning and a decrease in training-induced nuclear translocation of CaM and *c-Fos* expression, suggesting a role for NMDAR activation upstream to CaMKIIγ-mediated cytonuclear signaling in CaMKIIγ^–/–^ mice (Cohen et al., 2018). *In vivo* deletion of CaMKIIγ in parvalbumin (PV)-expressing inhibitory interneurons (CaMKIIγ PV-KO) eliminates NMDAR-induced synaptic potentiation of excitatory synapses onto inhibitory neurons (LTP_E→I_) and impairs experience-dependent neural oscillations, thereby disrupting memory consolidation and hippocampus-dependent LTM (He et al., 2021).

## Functional Implications of Ca^2+^/Calmodulin-Dependent Protein Kinase Type II Mutations in Diseases

In humans, *de novo* mutations in CaMKII have been identified and reported majorly in cases of neurodevelopmental disorders (NDDs) (Study, 2017; Akita et al., 2018) and intellectual disability (ID) (Küry et al., 2017). The role of CaMKII and glutamatergic signaling in neuropsychiatric diseases has been reviewed by Robison (Robison, 2014; see also Nicole and Pacary, 2020). Supplementary Table 2 summarizes the different CaMKII variants reported with their functional implications and clinical manifestations if any. The type of mutation (synonymous, missense, splice region, frameshift, deletion), the specific CaMKII isoform (α, β and γ) that is mutated and the protein domain (catalytic, auto-regulatory or association) affected determine the disease phenotype. The zygosity of inheritance (heterozygous/homozygous) can also influence the pathogenicity of the variant (Chia et al., 2018); however, intrafamilial variations in the expression of disease symptoms by subjects carrying the same heterozygous variant, have also been reported (Heiman et al., 2021).

Clinical manifestations of the identified mutations range from global neurodevelopmental delay, seizures, mild to severe ID, hypotonia, delayed development of motor and speech/language skills, abnormal emotional behavior, cerebellar atrophy, facial dysmorphism, visual impairment and gastrointestinal issues. Dysfunction of CaMKIIα can cause seizure-associated activity in the forebrain (Akita et al., 2018) and pronounced motor delay (Küry et al., 2017), while individuals with CaMKIIβ variants exhibit severe ID accompanied with hypotonia (Küry et al., 2017) and cerebellar atrophy (Akita et al., 2018). Facial dysmorphisms along with severe ID and severe hypotonia has been reported in patients carrying a *CAMK2G* variant (Proietti Onori et al., 2018). The vast majority of the variants identified, affect amino acids conserved across species (Küry et al., 2017; Stephenson et al., 2017; Akita et al., 2018; Chia et al., 2018; Proietti Onori et al., 2018), which may explain the degree of severity of pathogenicity.

Stephenson et al. (2017) reported the first characterization of a *de novo* missense mutation in the *CAMK2A* gene, encoding for CaMKIIα, that was found in a patient with autism spectrum disorder (ASD) (Iossifov et al., 2014). Replacement of Glu with Val at 183^rd^ position in the catalytic domain of CaMKIIα (CaMKIIα^Glu183Val^) disrupts the interaction of CaMKII with ASD-associated proteins, such as Shank3 (SH3 and multiple ankyrin repeat domains 3) (Jiang and Ehlers, 2013), GluN2B (Pan et al., 2015) and the metabotropic glutamate receptor mGlu5 (Chana et al., 2015), which can reduce targeting of CaMKIIα to spines (Stephenson et al., 2017). Neuronal expression of CaMKIIα^Glu183Val^ disrupts AMPAR-mediated synaptic transmission, interferes with CaMKII autophosphorylation and reduces dendritic spine density. Heterozygous (*Camk2a*^WT/E183V^) and homozygous (*Camk2a*^E183V/E183V^) KI mice displayed enhanced repetitive behaviors and deficits in social interactions, which mimic symptoms of ASD (Stephenson et al., 2017). Decreased autoinhibition and increased Thr^286^ autophosphorylation of the CaMKIIα^Pro212Gln^ mutant, identified in an individual with NDD, affects the efficiency of excitatory synaptic transmission by enhancing K^+^ currents in dendrites *in vitro* (Akita et al., 2018).

A biallelic, germline, loss-of-function *CAMK2A* missense mutation, *CAMK2A*^p.(His477Tyr)^ in the association domain of CaMKIIα, was reported in two siblings displaying psychomotor retardation, frequent seizures and severe ID (Chia et al., 2018). Compared to the WT enzyme, the mutant form disrupts CaMKIIα self-oligomerization and holoenzyme assembly which in turn affects its subcellular localization in neurons and ability to support synaptic function *in vivo* (Chia et al., 2018). Recently, Brown et al. (2021) characterized six heterozygous variants of *CAMK2A* found in patients with schizophrenia. The p.(Arg396*) mutation in the association domain of CaMKIIα ablates holoenzyme formation, impairs GluN2B binding and consequently fails to accumulate at excitatory synapses in response to a LTP stimulus. While both p.(Arg396*) and p.(Arg8His) variants of *CAMK2A* exhibited impaired autophosphorylation at Thr^286^, only the p.(Arg8His) mutation in the kinase domain significantly affected the Ca^2+^/CaM-stimulated kinase activity (Brown et al., 2021). The absence of impaired function or expression for the remaining four mutants studied indicates that the mere occurrence of a mutation in a patient does not imply that the disease is caused by the mutation (Brown et al., 2021).

In addition to NDDs and ID, *CAMK2A* variants/single nucleotide polymorphisms (SNPs)/single nucleotide variants (SNVs) have been reported to be associated with risk for bipolar disorder (BD) in cohorts of European descent (Ament et al., 2015), in sporadic AD patients belonging to the Han Chinese population (Fang et al., 2019) and mild cognitive impairment (MCI) subjects in a Spanish population (Bufill et al., 2015). Deletion of the chromosome at 5q32, covering *CAMK2A*, might be responsible for mild ID observed in two patients diagnosed with mandibulofacial dysostosis (Vincent et al., 2014). Interestingly, *CAMK2A* genetic variants have been reported to be nominally associated with non-verbal communication in ASD cohorts (Chiocchetti et al., 2018) and logical memory performance in the elderly people (Rhein et al., 2020). *CAMK2A* polymorphisms can also influence spatial working memory in Caucasian adolescents (Easton et al., 2013) and cognitive ability in Taiwanese senior high school students (Lee et al., 2021). Recruitment of higher number of subjects from distinct populations is warranted to further validate the association of *CAMK2A* SNPs in genotype-phenotype association studies (Chiocchetti et al., 2018; Rhein et al., 2020).

Apart from mutations in *CAMK2A*, *de novo* mutations in *CAMK2B* have also been reported in 10 unrelated individuals exhibiting mild to severe ID (Küry et al., 2017). There are 19 rare variants of *CAMK2A* and *CAMK2B* that are heterozygous nonsense, missense or splice-site mutations affecting the catalytic or auto-regulatory domain of CaMKII. The identified variants could affect protein expression and autophosphorylation at Thr^286^/Thr^287^ when expressed *in vitro*, and cause deficits in neuronal migration *in vivo* (Küry et al., 2017). *De novo* mutations in *CAMK2A* and *CAMK2B* can also result in varying neurodevelopmental phenotypes (Akita et al., 2018). The missense variants disrupted the interaction between the catalytic domain and the regulatory segment, leading to increased Ca^2+^-independent activity (Akita et al., 2018).

A heterozygous c.85C>T, p.(Arg29*) mutation in *CAMK2B* was found in a patient with mild ID, delayed speech development and seizures (Küry et al., 2017). This mutation was also reported in a 3-year-old European girl with complex focal seizures and global neurodevelopmental delay (Heiman et al., 2021). This maternally inherited pathogenic variant of the *CAMK2B* gene only mildly affected the patient’s sibling, with the same variant, while the mother was phenotypically healthy and intellectually normal (Heiman et al., 2021). Similarly, a heterozygous c.416C>T, p.(Pro139Leu) variant of *CAMK2B* found in four Caucasian patients presented with severe ID, global developmental delay, hypotonia and microcephaly (Küry et al., 2017), was also reported in a 22-year-old South Asian woman (Rizzi et al., 2020) as well as in a *MECP-2* (methyl-CpG binding protein 2) negative proband of Japanese origin (Iwama et al., 2019). The recurrence of a few pathogenic variants of *CAMK2A* and *CAMK2B* (Küry et al., 2017) calls for elaborate functional studies of the mutant proteins both *in vitro* and *in vivo* (Onori and van Woerden, 2021).

*De novo* mutations in *CAMK2G* have also been identified and reported in cases of NDDs and severe ID (De Ligt et al., 2012; Study, 2017; Proietti Onori et al., 2018). Whole-exome sequencing performed on two patients revealed c.875G>C, p.(Arg292Pro) mutation in the auto-regulatory domain of CaMKIIγ, which is a putative CaM trapping region. One research group showed that *CAMK2G*^p.(Arg292Pro)^ affects protein stability *in vitro* and functions as a pathogenic gain-of-function mutation by rendering it constitutively active and by blocking neuronal migration during development *in vivo* (Proietti Onori et al., 2018). The pathogenicity of the mutant is dependent on its catalytic activity (Proietti Onori et al., 2018). Cohen et al. (2018) reported that the ID observed in these patients might be due to the inability of CaMKIIγ^Arg292Pro^ to effectively trap CaM and shuttle Ca^2+^/CaM complex to the nucleus, thereby disrupting a major link connecting activation of NMDARs and Cav1 channels to nuclear transcription of *BDNF*, *c-Fos* and *Arc*. This in turn adversely affects synaptic strengthening and LTM *in vivo.* Similar to *CAMK2A* SNPs reported, a genetic cluster containing *CAMK2G* polymorphisms has been identified to be associated with episodic memory performance (Dominique and Papassotiropoulos, 2006).

Although the *CAMK2* variants reported so far shed light on the probable role of the kinase in mediating disease symptoms, the number of human subjects identified with the mutation is insufficient, compared to the samples tested, to correlate the variant to the disease with good statistical power. Neither is it mandatory for the identified variant(s) to be a causal factor in the diseased phenotype (Brown et al., 2021), nor can an indirect role by the mutant protein be overlooked. More detailed functional characterization of the identified and reported CaMKII mutations than what is already reported, both *in vitro* (Küry et al., 2017; Stephenson et al., 2017; Akita et al., 2018; Chia et al., 2018; Cohen et al., 2018; Proietti Onori et al., 2018; Brown et al., 2021) and *in vivo* (Stephenson et al., 2017; Chia et al., 2018), can further substantiate the critical role of CaMKII mutants in disease conditions. Nonetheless, screening for either sporadic or inherited *CAMK2* variants in disorders majorly affecting cognition, can help in unraveling the theragnostic potential of CaMKII, if any.

## Ca^2+^/Calmodulin-Dependent Protein Kinase Type II as a Druggable Target for Treating Glutamatergic Dysfunction

Antagonists against glutamate receptors, majorly NMDARs, have been designed, synthesized and evaluated for their efficacy in preventing excitotoxicity in CNS diseases (Liu et al., 2020; Chandran et al., 2021). Signaling molecules downstream to NMDARs, like CaMKII, can also be targeted to restore Ca^2+^ and glutamate homeostasis at synapses (Vest et al., 2010). Likewise, CaMKII has been exploited as a potential drug target in neuropsychiatric and neurodegenerative diseases (Sałaciak et al., 2021).

Based on the differential regulation of CaMKII function during neurotoxicity, the modulators either enhance (Yamamoto et al., 2009; Zeng et al., 2010; Wang D.M. et al., 2013; Wei et al., 2013; Wang S.Q. et al., 2014) or inhibit CaMKII activity (Wang D. et al., 2013; Jiang et al., 2019). Their *modus operandi* includes binding to Ca^2+^/CaM binding site of CaMKII (Brooks and Tavalin, 2011; Wong et al., 2019), targeting autonomous CaMKII activity (Coultrap et al., 2011; Wang D.M. et al., 2013; Wang et al., 2016; Deng et al., 2017), interacting with CaMKII hub domain (Leurs et al., 2021), preventing CaMKII translocation to the synaptic membrane (Matsumoto et al., 2008), inhibiting GluN2B-CaMKII binding (Tullis et al., 2021) or by modulating CaMKII-mediated signaling pathways (Liu et al., 2012; Matsumoto et al., 2013; Wei et al., 2013; Zhang et al., 2017; Islam et al., 2019; Wu et al., 2019; Izumi et al., 2020; Chen et al., 2021). The different CaMKII modulators reported from studies involving glutamatergic synapses in neurons are listed below:

1. Synthetic small molecule inhibitors like KN-62 and KN-93 (Tokumitsu et al., 1990; Sumi et al., 1991; Vest et al., 2010; Ashpole and Hudmon, 2011; Brooks and Tavalin, 2011).
2. Synthetic peptide inhibitors like AIP (autocamtide-2-related inhibitory peptide) (Fan et al., 2006; Goebel, 2009; Zha et al., 2009; Ashpole and Hudmon, 2011) and AC3-I (autocamtide-3 derived inhibitory peptide) (Leonard et al., 1999).
3. The natural CaMKII inhibitor protein CaM-KIIN (CN) and its peptide derivatives, CaM-KIINtide (CN27) (Chang et al., 1998; Saha et al., 2006; Mayadevi et al., 2016), CN21 (Vest et al., 2007, 2010; Ashpole and Hudmon, 2011; Ahmed et al., 2017), CN19 (Coultrap and Bayer, 2011; Chalmers et al., 2020) and CN17β (Gomez-Monterrey et al., 2013).
4. CaMKII antisense oligodeoxynucleotides (Liu et al., 2012).
5. Long non-coding RNA *CAMK2D*-associated transcript 1 (*C2dat1*) (Xu et al., 2016).
6. Analogs of γ-hydroxybutyrate (GHB) (Leurs et al., 2021).
7. Volatile anesthetics like isoflurane (Matsumoto et al., 2008).
8. Compounds isolated from natural sources like nobiletin (Yamamoto et al., 2009), curcumin (Mayadevi et al., 2012), β-asarone (Wei et al., 2013), paeoniflorin (Wang D. et al., 2013; Zhang et al., 2017), naringin (Wang D.M. et al., 2013), *Ganoderma lucidum* polysaccharides (GLP) (Wang S.Q. et al., 2014), baicalin (Wang et al., 2016), theobromine (Islam et al., 2019), tilianin (Jiang et al., 2019) and gastrodin (Chen et al., 2021).
9. The histone deacetylase (HDAC) inhibitor, vorinostat (Matsumoto et al., 2013).
10. SAK3, a T-type calcium channel enhancer (Izumi et al., 2020).

Although a majority of the modulators have been widely employed to combat neuronal glutamatergic dysfunction *in vitro* and *in vivo*, their clinical use will require extensive studies on possible side effects and toxicity to cardiac health too (Nassal et al., 2020).

## Future Perspectives

Majority of the biochemical and structural studies on CaMKII have been performed on homomeric holoenzymes of one of the isoforms. However, under physiological conditions the enzyme can form hetero-multimers with different subunit stoichiometries. The relative abundance of different heteromeric subtypes under different developmental stages and in different regions would be an important determinant in the physiological functioning of CaMKII. Detailed studies on the functional variation among heteromers and their participation in specific cellular functions would be essential in making further progress in understanding the physiological functions of CaMKII. Further refinement of molecular genetic techniques for ectopic expression of isoforms and mutants of CaMKII with better control on heteromer formation would be essential for progress toward this goal.

The binding of CaMKII to GluN2B modulates the kinetic parameters of CaMKII enzyme activity and attenuates dephosphorylation of CaMKII (Pradeep et al., 2009; Cheriyan et al., 2011). These regulatory events strongly support the bistable switch model of molecular memory involving CaMKII and PP1. However, the physiological relevance of these regulatory events and the existence of the bistable switch *in vivo* needs to be demonstrated. Elucidation of the structure of the CaMKII-GluN2B complex could contribute significantly toward understanding the physiological functions of this complex.

CaMKII activation is a prerequisite for both LTP and LTD and recently it has been shown (Woolfrey et al., 2018) that two types of substrates (high autonomy, low autonomy) are preferred under each condition. If so, what are the exact signaling events with respect to the amount of Ca^2+^ entry into the postsynaptic site responsible for each of these events?

The recurrence of a few pathogenic variants of *CAMK2A* and *CAMK2B* (Küry et al., 2017) calls for elaborate functional studies of the mutant proteins both *in vitro* and *in vivo* (Onori and van Woerden, 2021). It also encourages more extensive screening for genetic variants of CaMKII in human populations.

## Conclusion

CaMKII is an enzyme highly enriched in the brain. It has important roles to play in the functioning of glutamatergic synapses. Significant advances have been made in understanding the structure, function and physiological role of CaMKII. Its contribution to learning and memory has been investigated extensively with the help of most modern techniques. This has unequivocally established the integral part played by this enzyme in learning and memory. However, there is still more to be understood about the exact manner in which CaMKII participates in the underlying cellular mechanisms such as synaptic plasticity. Novel features of the structure and biochemical regulation of CaMKII are still being revealed by biophysical and biochemical experiments. Since the molecular properties of CaMKII holoenzyme are among the foundations on which most of the models of synaptic plasticity, learning and memory are built, progress in structural studies would continue to necessitate revisions in these models. Powerful molecular genetic techniques have permitted the controlled expression of isoforms and mutants of CaMKII in specific cell types in the brain of model organisms leading to important insights into its role in the cellular and systemic mechanisms. However, it has been difficult to dissect out all of its synaptic functions from cellular functions due to technical hurdles. The occurrence of heteromeric subtypes of CaMKII and the redundancy in the function among the isozymes also poses challenges to molecular genetic interrogation of its cellular functions. Progress in the understanding of CaMKII has prompted attempts to pursue it as a therapeutic target for pharmacological and genetic interventions since it is part of impaired Ca^2+^ signaling in many disease conditions. A limited number of genetic variants of CaMKII have been found associated with human neurological disease conditions. The central role of CaMKII in brain functions calls for large scale screening for CaMKII variants in human populations.

## Author Contributions

AM and RO contributed to conception and design of the manuscript. AM, SG, RJ, and RO co-wrote the manuscript. All authors contributed to manuscript revision, read and approved the submitted version.

## Conflict of Interest

The authors declare that the research was conducted in the absence of any commercial or financial relationships that could be construed as a potential conflict of interest.

## Publisher’s Note

All claims expressed in this article are solely those of the authors and do not necessarily represent those of their affiliated organizations, or those of the publisher, the editors and the reviewers. Any product that may be evaluated in this article, or claim that may be made by its manufacturer, is not guaranteed or endorsed by the publisher.
