# Supplementary material for: Role of Ca2+/Calmodulin-Dependent Protein Kinase Type II in Mediating Function and Dysfunction at Glutamatergic Synapses
Source: Front Mol Neurosci. 2022 Jun 20;15:855752. doi: 10.3389/fnmol.2022.855752 (PMC9252440; doi:10.3389/fnmol.2022.855752)
Supplement: Supplementary file 1 [file Table_1.pdf]

Supplementary Table 1: Synaptic substrates of CaMKII

Sl. No.	Name of the substrate	CaMKII phosphorylation site(s)	Functional relevance	Reference
1	CaMKII	$Thr^{286/287}$	LTP	Lisman and Zhabotinsky, 2001
2	CaMKII	$Thr^{305/306}$	Inhibits CaMKII function	Vest et al., 2007
3	NMDAR subunit GluN2B	Ser^{1303}	Regulates the interaction with CaMKII	McGlade-McCulloh et al., 1993
4	NMDAR subunit GluN2A	Ser^{1291}	NMDAR current potentiation	Chen et al., 2007
5	AMPAR subunit GluA1	Ser^{831}	Enhances AMPA channel conductance; LTP	Derkach et al., 1999
6	PSD95; PDZ1 domain	Ser^{73}	Facilitates dissociation of GluN2A from PSD95	Gardoni et al., 2006
7	Synapse-associated protein (SAP) 97	Ser^{39}	GluA1 redistribution in AMPAR, SAP97 trafficking to spines	Mauceri et al., 2004
8	SAP97	Ser^{232}	Modulates binding of other proteins, Disrupts interaction with GluN2A	Gardoni et al., 2003; Nikandrova et al, 2010
9	TARPγ-8	Ser^{277}, Ser^{281}	Role in synaptic plasticity; Enhances synaptic insertion of AMPAR	Park et al., 2016
10	Arc	Ser^{260}	Regulates oligomerization of Arc; Synaptic plasticity	Zhang et al., 2019
11	Guanylate kinase-associated protein (GKAP)	Ser^{54}	Induces poly ubiquitination of GKAP	Shin et al., 2012
12	Densin-180	Ser^{94}, Ser^{134}	Regulates binding with other proteins in PSD95	Strack et al., 2000b
13	SynGAP synaptic Ras/Rap	Ser^{1123}, Ser^{1058}, $Ser^{750/751/756}$, $Ser^{764/765}$	Increases RasGTPase activity	Oh et al., 2004

	GTPase-activating protein			
14	Vimentin	Ser^{82}	Synaptic reorganization	Inagaki et al., 1997
15	Stargazin (TARPγ-2)	Ser^{9}	Stops the diffusion of surface AMPARs at synaptic sites; Promotes AMPAfication	Opazo et al., 2010
16	Nav1.6	Ser^{561} and Ser^{641}/Thr^{642}	Allows Na^{+} influx and helps in neuronal excitability	Zybura et al., 2020
17	AKAP79/150	Thr^{87}, Ser^{92}	Spine shrinkage; LTD	Woolfrey et al., 2018
18	Kalirin 7	Thr^{95}	Activation of PKA; AMPAfication; Spine enlargement	Xie et al., 2007
