# Supplementary material for: Role of Ca2+/Calmodulin-Dependent Protein Kinase Type II in Mediating Function and Dysfunction at Glutamatergic Synapses
Source: Front Mol Neurosci. 2022 Jun 20;15:855752. doi: 10.3389/fnmol.2022.855752 (PMC9252440; doi:10.3389/fnmol.2022.855752)
Supplement: Supplementary file 2 [file Table_2.pdf]

Supplementary Table 2: Summary of CaMKII variants reported in human diseases

***CAMK2* Gene (CaMKII protein isoform)**	**Identified variant**			**Functional implication(s)**	**Clinical manifestation (s)**	**Reference(s)**
	**Genomic DNA (SNP)[a]**	**Coding DNA[b]**	**Protein[c]**			
CAMK2A (CaMKIIα)	g.149669166C>T	c.23G>A	p.(Arg8His)	↓ pT^{286}; ↓ Ca^{2+}/CaM-stimulated activity	SCZ	Brown et al., 2021
	g.150281027T>A or g.150281027T>C or g.150281027T>G (rs6881743)	--	--	Intron variant	AD	Fang et al., 2019
	g.150276863C>T (rs10515639)	--	--	Intron variant	AD	Fang et al., 2019
	g.149652720del	c.65del	p.(Gly22Glufs*10)	Truncated, non-functional protein	Moderate ID	Küry et al., 2017
	g.150261592G>A (rs3822606)	--	--	Intron variant	AD/MCI	Bufill et al., 2015
	g.150258797G>A (rs4958445)	--	--	Intron variant	AD	Fang et al., 2019
	g.149636374A>G	c.293T>C	p.(Phe98Ser)	↓ pT^{286}; ↓ neuronal migration	Moderate ID	Küry et al., 2017
	g.149636340C>G	c.327G>C	p.(Glu109Asp)	↑ pT^{286}; ↓ neuronal migration	Severe ID, DD	Küry et al., 2017; Study, 2017
	g.149636332G>A	c.335C>T	p.(Ala112Val)	--	Severe ID	Küry et al., 2017
	g.149633109G>C	c.412C>G	p.(Pro138Ala)	No effect observed	DD	Küry et al., 2017; Study, 2017
	g.149631595T>A	c.548A>T	p.(Glu183Val)	Unstable protein; ↓ pT^{286}; ↓ neuronal migration; ↓ spine density; ↑ dendritic arborization; disruption of excitatory synaptic transmission	ASD, mild ID	Iossifov et al., 2014; Küry et al., 2017; Stephenson et al., 2017
	g.149631543dup	c.598+2dup	Skipping of in-frame exon 8	Partial loss of catalytic domain	ASD, severe ID	Iossifov et al., 2014; Küry et al., 2017
	g.149631371G>A	c.635C>T	p.(Pro212Leu)	Uncertain	Mild to severe ID	Küry et al., 2017

	g.149631371G>T	c.635C>A	p.(Pro212Gln)	Disruption of catalytic-autoregulatory domain interaction; ↑ pT^{286}	NDD, seizures	Akita et al., 2018
	g.149630365C>T	c.702G>A	Silent variant	--	DD	Study, 2017
	g.149630363G>A	c.704C>T	p.(Pro235Leu)	Disruption of catalytic-autoregulatory domain interaction	Mild ID, NDD, seizures	Küry et al., 2017; Akita et al., 2018
	g.149629873C>T	c.817-1G>A	p.His273_Lys300del	Loss of autoinhibitory domain	Severe ID, NDD, seizures	Küry et al., 2017; Akita et al., 2018
	g.149629844T>C	c.845A>G	p.(His282Arg)	Unstable protein, ↑ pT^{286}; ↓ neuronal migration	Severe ID	Küry et al., 2017
	g.149629833T>G	c.856A>C	p.(Thr286Pro)	No pT^{286}; Blockage of neuronal migration	Severe ID	Küry et al., 2017
	g.150249081C>T or g.150249081C>G (rs3756577)	--	--	Intron variant	AD/MCI	Bufill et al., 2015; Fang et al., 2019
	g.150239936G>A or g.15029936G>C (rs3797617)	--	--	Intron variant	AD	Fang et al., 2019
	g.150237156G>A or g.150237156G>C (rs6869634)	--	--	Intron variant	AD	Fang et al., 2019
	g.14960773G>A	c.1186C>T	p.(Arg396*)	↓ holoenzyme assembly; ↓ pT^{286}; ↓ GluN2B binding	SCZ	Brown et al., 2021
	g.149607754C>T	c.1204+1G>A	Skipping of out-of-frame exon 17	Replacement of association domain by a 178 aa long tail	Mild ID, DD	Küry et al., 2017; Study, 2017
	g.150227185T>C (rs10051644)	--	--	Intron variant	AD	Fang et al., 2019
	g.149602688G>A	c.1297C>T	p.(Arg433Cys)	No effect observed	SCZ	Brown et al., 2021
	g.149602589G>A	c.1429C>T	p.(His477Tyr)	Disrupts self-oligomerization and holoenzyme assembly	Severe ID, seizures, developmental delay	Chia et al., 2018

	g.149602555G>T	c.1430C>A	p.(Pro477His)	No effect observed	SCZ	Brown et al., 2021
CAMK2B (CaMKIIβ)	g.44323805G>A	c.85C>T	p.(Arg29*)	Truncated and non-functional protein	Mild ID, seizures, global neurodevelopmental delay	Küry et al., 2017; Heiman et al., 2018
	g.44294154C>T	c.328G>A	p.(Glu110Lys)	Unstable protein; ↑ pT^{287}; ↓ neuronal migration	Mild ID	Küry et al., 2017
	g.44283125G>A	c.416C>T	p.(Pro139Leu)	Unstable protein; ↑ pT^{287}; ↓ neuronal migration	Severe ID, DD, epilepsy, cerebellar atrophy	Küry et al., 2017; Study, 2017; Rizzi et al., 2020; Iwama et al., 2019
	g.44282257C>A	c.602-4G>T	Splice region variant	--	DD	Study, 2017
	g.44282217G>A	c.638C>T	p.(Pro213Leu)	Disrupt catalytic-autoregulatory domain interaction	NDD, cerebellar atrophy	Akita et al., 2018
	g.44282206C>T	c.649G>A	p.(Glu217Lys)	--	Unaffected	Iossifov et al., 2014
	g.44281927C>T	c.709G>A	p.(Glu237Lys)	↑ pT^{287}; ↓ neuronal migration	Severe ID, DD	Küry et al., 2017; Study, 2017
	g.44281383C>T	c.820-1G>A	Skipping of in-frame exon 11	Loss of autoinhibitory domain	Severe ID	Küry et al., 2017
	g.44281350T>A	c.852A>T	p.(Arg284Ser)	Disrupt interaction between catalytic-autoregulatory domains	NDD, cerebellar atrophy	Akita et al., 2018
	g.44281301T>C	c.901A>G	p.(Lys301Glu)	No pT^{287}	Severe ID	Küry et al., 2017
	g.44281298C>T	c.903+1G>A	Skipping of in-frame exon 11	Loss of autoinhibitory domain	Mild to severe ID	Küry et al., 2017
	g.44266243G>T	c.1469C>A	p.(Ser490Tyr)	--	DD	Study, 2017
CAMK2G (CaMKIIγ)	g.75602244C>G	c.875G>C	p.(Arg292Pro)	Unstable protein; ↑ pT^{287}; ↓ neuronal migration; ↓ neurite length; ↓ CaM trapping; affects LTM	Severe ID, severe hypotonia, facial dysmorphism	De Ligt et al., 2012; Proietti Onori et al., 2018; Cohen et al., 2018
	g.75587858C>T	c.1075G>A	p.(Val359Met)	--	DD	Study, 2017

[a]Reference genome used is NC_000005.9 (Iossifov et al., 2014; Küry et al., 2017; Study, 2017; Akita et al., 2018; Chia et al., 2018; Brown et al., 2021), NC_000005.10 (Bufill et al., 2015; Fang et al., 2019) for *CAMK2A*,

NC_000007.13 (Iossifov et al., 2014; Küry et al., 2017; Study, 2017; Akita et al., 2018) for *CAMK2B* and NC_000010.10 (De Ligt et al., 2012; Study, 2017) for *CAMK2G*

[b]Transcript reference sequence used is NM_171825.2 (Küry et al., 2017; Study, 2017; Brown et al., 2021), NM_015981.3 (Akita et al., 2018) for *CAMK2A*, NM_172079.2 (Iossifov et al., 2014; Küry et al., 2017), NM_001220.4 (Study, 2017; Akita et al., 2018) for *CAMK2B* and NM_172171.2 (De Ligt et al., 2012), NM_172169.2 (Study, 2017) for *CAMK2G*

[c]Protein reference sequence used is NP_741960.1 (Küry et al., 2017; Study, 2017; Brown et al., 2021), NP_057065.2 (Akita et al., 2018), Q9UQM7-2 (Chia et al., 2018) for CaMKIIα, NP_742076.1 (Iossifov et al., 2014; Küry et al., 2017), NP_001211.3 (Study, 2017; Akita et al., 2018) for CaMKIIβ and NP_751911.1 (De Ligt et al., 2012), NP_751909.1 (Study, 2017) for CaMKIIγ

Abbreviations: AD-Alzheimer's Disease, DD-Developmental Disorder, ID-Intellectual Disability, MCI-Mild Cognitive Impairment, NDD-Neurodevelopmental Disorder, SCZ-Schizophrenia, SNP-Single Nucleotide Polymorphism
